# Endocannabinoid modulation of defensive state transitions to innate and learned threat

**DOI:** 10.1007/s00213-025-06812-z

**Published:** 2025-05-24

**Authors:** Niharika Loomba, Anyu Cao, Senna Charles, Isaac Kandil, Michelle Kwon, Sachin Patel

**Affiliations:** 1 Vanderbilt Brain Institute, Vanderbilt University, Nashville, TN USA; 2 Department of Psychiatry and Behavioral Sciences, Stephen M. Stahl Centre for Psychiatric Neuroscience, Northwestern University Feinberg School of Medicine, Chicago, IL USA; 3 Neuroscience Graduate Program, University of California, San Francisco, CA USA

**Keywords:** Endocannabinoids, 2-AG, AEA, Conditioned fear, Innate fear, Looming shadow

## Abstract

A hallmark of many psychiatric disorders is maladaptive and heightened fear responses to non-threatening stimuli. Adaptive defensive responses to threats involve transitions between passive behaviors, such as freezing, and active escape strategies, such as darting or fleeing. The endocannabinoid (eCB) system, particularly 2-arachidonoylglycerol (2-AG), plays a crucial role in modulating fear and stress responses. However, the extent to which 2-AG influences defensive behavioral state transitions to fear responses remains unclear. To address this, we investigated the role of 2-AG in shaping defensive behaviors to learned and innate threats using pharmacological manipulations in both the serial compound stimulus (SCS) and the looming shadow paradigm. During SCS, inhibition of 2-AG synthesis enhanced freezing to early cues and promoted active responses during cues associated with heightened threat imminence. In the looming shadow paradigm, 2-AG depletion biased defensive behavior toward freezing and increased time spent in a safe zone, suggesting a shift toward passive responses. These findings demonstrate that 2-AG signaling critically regulates the balance and transitions between passive and active defensive strategies in both learned and innate fear contexts. Thus, 2-AG plays a key role in the scaling of defensive response transitions and the promotion of active defensive responses to threats.

**Supplementary Information:**

The online version contains supplementary material available at 10.1007/s00213-025-06812-z.

## Introduction

The selection of appropriate defensive responses to actual and perceived threats is vital to an organism’s survival (Fadok et al. 2017; Borkar et al. 2020; Le et al. 2024). However, heightened fear responses in non-threatening situations are a hallmark symptom of anxiety- and stress-related disorders. According to the predatory imminence theory, defensive behaviors shift as the proximity of a threat, both spatially and temporally, increases (Fanselow and Lester 1988; Blanchard and Blanchard 1989; Fanselow 2018). Elucidating the neurobiological mechanisms underlying these adaptive behaviors could be important for uncovering potential therapeutic targets for these highly prevalent mental disorders.

The endocannabinoid (eCB) system is a neuromodulatory system that functions to suppress neurotransmission. 2-arachidonylglycerol (2-AG), the brain’s most abundant eCB, is synthesized in the postsynaptic membrane in an activity-dependent manner by the enzyme diacylglycerol lipase alpha (DAGLα). It then retrogradely activates G_*i/o*_-coupled cannabinoid receptor 1 (CB1R) on presynaptic terminals to inhibit neurotransmitter release before being degraded by monoacylglycerol lipase (MAGL) (Kano et al. 2009). A large body of work has demonstrated the central role of 2-AG in responding to stress and threat (Riebe et al. 2012; Lutz et al. 2015; Patel et al. 2022; Gunduz-Cinar et al. 2023; Ramos-Medina et al. 2024). Additionally, augmentation of anandamide (AEA), another common eCB molecule, signaling has shown anxiolytic effects in response to stress and facilitation of fear extinction (Kathuria et al. 2003; Lafenêtre et al. 2007; Gunduz-Cinar et al. 2013; Petrie et al. 2021). However, the role of the eCB system in modulating transitions between defensive states across the spectrum of threat imminence remains elusive.

To assess learned defensive response switching, we used the serial compound stimulus (SCS) paradigm that elicits freezing followed by flight (darting/jumping) responses as the auditory stimulus progresses from tone to white noise. Specifically, SCS consisted of a 10-s long pure tone followed immediately by 10 s of white noise and terminates with a strong 1-s foot shock (Fadok et al. 2017; Borkar et al. 2020; Totty et al. 2021; Le et al. 2024). This protocol allowed us to evaluate behavioral state transitions as threat imminence and salience increase during the transition from pure tone to white noise (Fadok et al. 2017). To explore innate defensive response switching we used a looming shadow paradigm (Daviu et al. 2020a, 2020b; Gunduz-Cinar et al. 2013; Shang et al. 2018; Yilmaz & Meister 2013). In this paradigm, mice are repeatedly exposed to growing overhead shadows to mimic descent by an aerial predator, to examine defensive responses spanning from freezing to the shadow to fleeing to a safe space (covered tent).

By combining these behavioral paradigms with pharmacological manipulation of 2-AG levels, this study provides insight into the role of the eCB system in mediating defensive responses across a spectrum of learned and innate threats. We hypothesize that 2-AG signaling critically influences the selection and transition between passive and active coping strategies during escalating threat imminence. Understanding how the eCB system modulates these behaviors can deepen our knowledge of the neurobiological mechanisms underlying adaptive and maladaptive responses to fear and stress, ultimately guiding the development of targeted therapies for anxiety- and stress-related disorders.

## Materials and methods

### Animal care

Male and female C57BL/6 J mice aged 8–12 weeks were used for all behavioral experiments. Animals were group-housed (five animals per cage) on a 12-h light/dark cycle with food and water provided ad libitum. Experiments were conducted during the light phase. Mice were given a one-week acclimation period to the facilities and handled by experimenters before testing. All experiments were carried out according to guidelines provided by Northwestern University Animal Care and Use Committee.

### Drug treatment

All treatments were injected intraperitoneally (I.P.) 2 h before testing on conditioning days for the fear conditioning paradigms and 2 h before looming shadow testing. DO34 (50 mg/kg, Glixx Laboratories) was dissolved in an 18:1:1 solution of saline (Hospira, Inc.), ethanol (Decon Laboratories, Inc.), and kolliphor EL (Sigma-Aldrich). Vehicle treatment consisted of the 18:1:1 solution only. JZL-184 (10 mg/kg, Cayman Chemical Company) or PF-3845 (1 mg/kg, Cayman Chemical Company) were dissolved in 100% dimethylsulfoxide (DMSO; 1 μl/g, Sigma-Aldrich), with 100% DMSO as the corresponding vehicle (Morgan et al. 2022; Kondev et al. 2022, 2023a).

### SCS paradigm

The serial compound stimulus (SCS) conditioned flight protocol was used as previously described (Fadok et al. 2017). Two contexts were used for the SCS paradigm. Context A was a half-circle chamber made of a multi-colored plexiglass wall and floor. The behavior room lights were dimmed. Red tape was added to lights within the chamber to alter the lighting, and a vanilla scent was used to further distinguish Context A. Context B was a rectangular chamber (38 cm x 19 cm x 30 cm) with metal floor grids housed within soundproof boxes (Coulbourn Instruments). 70% ethanol was used to clean both contexts. Auditory stimuli were delivered at 75 dB via speakers within the boxes above the chambers and foot shocks were delivered via the metal grids using FreezeFrame software (Actimetrics). On all days, animals were given a 3-min habituation period to the context before the onset of the first auditory stimulus. On Day 0, four SCS pairings of 10 s pure tone (7.5 kHz) pips (500 ms at 1 Hz) and 10 s white noise pips (500 ms at 1 Hz) were delivered in context A with pseudorandom intertrial intervals (ITIs) of 50–90 s. On Days 1 and 2, five pairings of SCS were delivered in context B with pseudorandom ITIs of 150–210 s, followed immediately by a 1 s shock (0.9 mA). On Day 3, sixteen pairings of SCS were delivered in context B with pseudorandom ITIs of 60–100 s for fear extinction.

### Escape score calculations

The escape score was calculated as a combination of speed differences between experimental phases and the number of jumps observed during each phase. First, the mean speed during the Pre-SCS (baseline), Pure Tone (PT), and White Noise (WN) phases was computed for each trial. To stabilize variance, all speed values were transformed using a natural logarithm, with a small constant (epsilon) added to avoid undefined values. The speed difference for each phase was then calculated by subtracting the log-transformed Pre-SCS speed from the log-transformed speeds during the PT and WN phases, reflecting changes in activity relative to baseline. The number of jumps during the PT and WN phases was directly added to their respective speed differences to account for escape-related motor events. This approach yielded escape scores that integrate both locomotor and discrete escape behaviors.

1. Speed Data Preparation:
  Pre-SCS period (Pre-SCS): The mean speed during the −10 to 0 seconds interval.
  Pre-SCS period (Pre-SCS): The mean speed during the −10 to 0 seconds interval.
  Pure Tone (PT) period: The mean speed during the 1 to 10 s interval.
  White Noise (WN) period: The mean speed during the 11 to 20 s interval.
2. Log Transformation: applied to speed values to reduce skewness of data.
  LogSpeed_Pre-SCS_ = ln(Speed_Pre-SCS_ + ϵ)
  LogSpeed_PT_ = ln(Speed_PT_ + ϵ)
  LogSpeed_WN_ = ln(Speed_WN_ + ϵ)
3. Speed Difference Calculation:
  SpeedDiff_PT_= LogSpeed_PT_ - LogSpeed_Pre-SCS_
  SpeedDiff_WN_= LogSpeed_WN_ - LogSpeed_Pre-SCS_
4. Escape Score Calculation.
  EscapeScore_PT_ = SpeedDiff_PT_ + Jumps_PT_
  EscapeScore_WN_ = SpeedDiff_WN_ + Jumps_WN_

### Looming shadow paradigm

Behavior was tested in a clear rectangular arena (40 cm × 20 cm × 16 cm) with a red plexiglass shelter on one end of the space. A red light was placed outside of the arena on the tent side to see the mouse inside the tent. A rear-end projection screen was suspended 30 cm above the base of the arena and a projector (Elephas) sat 66 cm above the screen to provide projection of the looming shadow stimuli. A separate computer displaying the looming shadow stimuli was connected to the projector. Shadow stimuli consisted of five cycles of a black circle growing over 500 ms, with the total duration lasting 3 s. Stimuli were triggered manually by the experimenter when the animal entered a predefined area on the opposite end of the cage from the shelter. Animal behavior was recorded using ANY-maze.

Subjects were given 15 min to habituate to the arena prior to the onset of the looming shadow stimuli. The animals then underwent a 15-min testing stage, in which they were presented with a shadow when the animal entered the pre-determined “far zone” (20 cm × 8 cm) on the opposite end of the arena. After the shadow presentation, a one-minute inter-presentation interval was implemented during which no shadow stimuli were triggered, irrespective of the animal's location. Testing was concluded after the subject executed five shadow presentations or upon the expiration of the 15-min testing period. 70% ethanol was used to clean the arena between animals.

### Behavioral analyses

For fear conditioning, freezing behavior was quantified using Freezeframe, and animal speed was quantified using ANY-maze with the animal’s centroid tracked for analysis purposes. All behaviors were binned into 1 s bins. An independent investigator blind to treatment hand-scored all jump data.

For looming shadow, an independent investigator blind to treatment hand-scored the length of time an animal spent in the tent and the latency to respond to each shadow. Defensive responses were categorized as no response, freeze, dart (fleeing to tent immediately after shadow presentation), freeze-to-dart (freezing then fleeing to the tent), or timed out (were unable to receive all five shadow responses in the 15-min testing period due to excessive time in the tent).

### Statistical analyses

All statistical analyses were performed with Prism 10 (GraphPad, San Diego, CA, USA) and MATLAB R2024a (MathWorks, Natick, MA, USA) software. Normality was determined using the Shapiro–Wilk test. Group effects were analyzed using paired Student’s t-test or analysis of variance (ANOVA), depending on the number of independent variables. When correcting for multiple comparisons, post-hoc analyses of ANOVAs included Holm-Šídák's or Dunnett’s test. Otherwise, Fisher's Least Significant Difference (LSD) was used. Chi-squared analyses were conducted in MATLAB. Details for each analysis can be found in Tables 1, 2, 3, 4, 5, 6, and 7. *P* < 0.05 was considered significant throughout.

**Table 1:** Statistical Analyses for SCS Conditioning days with DO34 and Vehicle treatments

Figure	Statistical Test	Post-hoc Analysis	Main Effects	Interactions
2A	2-way RM ANOVA	Sidak's multiple comparisons	Stimulus: F (40, 320) = 8.719, *P* < 0.0001	F (40, 320) = 1.755, *P* = 0.0046
			Treatment: F (1, 8) = 0.2010, *P* = 0.6658	
2B	2-way RM ANOVA	Sidak's multiple comparisons	Stimulus: F (1, 16) = 73.28, *P* < 0.0001	F (1, 16) = 13.11, *P* = 0.0023
			Treatment: F (1, 16) = 0.001781, *P* = 0.9669	
2C	2-way RM ANOVA	Sidak's multiple comparisons	Stimulus: F (3.845, 30.76) = 84.45, *P* < 0.0001	F (39, 312) = 2.192, *P* = 0.0001
			Treatment: F (1, 8) = 0.6337, *P* = 0.4490	
2D	2-way RM ANOVA	Sidak's multiple comparisons	Stimulus: F (1, 16) = 20.43, *P* = 0.0003	F (1, 16) = 9.608, *P* = 0.0069
			Treatment: F (1, 16) = 0.1134, *P* = 0.7407	
2E	3-way ANOVA	N/A	Trials: F (4, 64) = 2.861, *P* = 0.0303	Trials x Treatment: F (4, 64) = 2.686, *P* = 0.0390 Trials x Stimulus: F (4, 64) = 2.118, *P* = 0.0888 Treatment x Stimulus: F (1, 16) = 11.26, *P* = 0.0040 Trials x Treatment x Stimulus: F (4, 64) = 3.588, *P* = 0.0106
			Treatment: F (1, 16) = 7.902, *P* = 0.0125	
			Stimulus: F (1, 16) = 11.26, *P* = 0.0040	
2F	2-way RM ANOVA	Fisher's LSD	Stimulus: F (1, 16) = 11.26, *P* = 0.0040	F (1, 16) = 11.26, *P* = 0.0040
			Treatment: F (1, 16) = 7.902, *P* = 0.0125	
2G	2-way RM ANOVA	Fisher's LSD	Stimulus: F (1, 16) = 36.81, *P* < 0.0001	F (1, 16) = 9.582, *P* = 0.0069
			Treatment: F (1, 16) = 1.277, *P* = 0.2751	
2H	2-way RM ANOVA	Sidak's multiple comparisons	Stimulus: F (4.994, 39.95) = 42.59, *P* < 0.0001	F (40, 320) = 2.106, *P* = 0.0002
			Treatment: F (1, 8) = 32.47, *P* = 0.0005	
2I	2-way RM ANOVA	Sidak's multiple comparisons	Stimulus: F (1, 16) = 220.4, *P* < 0.0001	F (1, 16) = 9.533, *P* = 0.0071
			Treatment: F (1, 16) = 10.61, *P* = 0.0049	
2J	2-way RM ANOVA	Sidak's multiple comparisons	Stimulus: F (4.752, 38.01) = 37.38, *P* < 0.0001	F (39, 312) = 3.602, *P* < 0.0001
			Treatment: F (1, 8) = 6.234, *P* = 0.0371	
2K	2-way RM ANOVA	Sidak's multiple comparisons	Stimulus: F (1, 16) = 77.87, *P* < 0.0001	F (1, 16) = 12.63, *P* = 0.0026
			Treatment: F (1, 16) = 2.314, *P* = 0.1477	
2L	3-way ANOVA	N/A	Trials: F (2.698, 43.17) = 3.407, *P* = 0.0298	Trials x Treatment: F (4, 64) = 0.2169, *P* = 0.9281 Trials x Stimulus: F (2.153, 34.44) = 5.347, *P* = 0.0082 Treatment x Stimulus: F (1, 16) = 9.490, *P* = 0.0072Trials x Treatment x Stimulus: F (4, 64) = 0.5252, *P* = 0.7176
			Treatment: F (1, 16) = 5.465, *P* = 0.0327	
			Stimulus: F (1.000, 16.00) = 29.29, *P* < 0.0001	
2M	2-way RM ANOVA	Fisher's LSD	Stimulus: F (1, 16) = 32.12, *P* < 0.0001	F (1, 16) = 7.643, *P* = 0.0138
			Treatment: F (1, 16) = 4.167, *P* = 0.0581	
2N	2-way RM ANOVA	Fisher's LSD	Stimulus: F (1, 16) = 83.52, *P* < 0.0001	F (1, 16) = 12.60, *P* = 0.0027
			Treatment: F (1, 16) = 0.0004679, *P* = 0.9830

**Table 2:** Statistical Analyses for SCS Extinction with DO34 and Vehicle treatments

Figure	Statistical Test	Post-hoc Analysis	Main Effects	Interactions
3A	2-way RM ANOVA	Sidak's multiple comparisons	Stimulus: F(6.527, 195.8) = 13.92, *P* < 0.0001	F (40, 1200) = 1.533, *P* = 0.0188
			Treatment: F (1, 30) = 11.39, *P* = 0.0021	
3B	2-way RM ANOVA	Sidak's multiple comparisons	Stimulus: F (1, 16) = 73.94, *P* < 0.0001	F (1, 16) = 0.2341, *P* = 0.6351
			Treatment: F (1, 16) = 1.552, *P* = 0.2308	
3C	2-way RM ANOVA	Sidak's multiple comparisons	Stimulus: F (6.527, 195.8) = 13.92, *P* < 0.0001	F (39, 1170) = 1.209, *P* = 0.1790
			Treatment: F (1, 30) = 2.669, *P* = 0.1128	
3D	2-way RM ANOVA	Sidak's multiple comparisons	Stimulus: F (1, 16) = 71.37, *P* < 0.0001	F (1, 16) = 0.7362, *P* = 0.4035
			Treatment: F (1, 16) = 1.894, *P* = 0.1877	
3E	3-way ANOVA	Tukey's multiple comparisons	Trials: F (3, 45) = 24.74, *P* < 0.0001	Trials x Treatment: F (3, 45) = 2.927, *P* = 0.0438 Trials x Stimulus: F (3, 45) = 24.12, *P* < 0.0001 Treatment x Stimulus: F (1, 15) = 4.459, *P* = 0.0519 Trials x Treatment x Stimulus: F (3, 45) = 1.104, *P* = 0.3572
			Treatment: F (1, 15) = 1.607, *P* = 0.2243	
			Stimulus: F (1, 15) = 19.94, *P *= 0.0005	
3F	2-way RM ANOVA	Sidak's multiple comparisons	Stimulus: F (1, 16) = 82.40, *P* < 0.0001	F (1, 16) = 5.440e-006, *P* = 0.9982
			Treatment: F (1, 16) = 0.4634, *P* = 0.5058	
3G	2-way RM ANOVA	Sidak's multiple comparisons	Stimulus: F (1, 16) = 142.2, *P* < 0.0001	F (1, 16) = 1.457, *P* = 0.2449
			Treatment: F (1, 16) = 4.364, *P* = 0.0530	
3H	2-way RM ANOVA	Fisher's LSD	Stimulus: F (1, 15) = 36.61, *P* < 0.0001	F (1, 15) = 2.352, *P* = 0.1460
			Treatment: F (1, 15) = 4.250, *P* = 0.0570	
3I	2-way RM ANOVA	Fisher's LSD	Stimulus: F (1, 16) = 105.6, *P* < 0.0001	F (1, 16) = 0.05035, *P* = 0.8253
			Treatment: F (1, 16) = 4.220, *P* = 0.0567	
3J	2-way RM ANOVA	Sidak's multiple comparisons	Stimulus: F (1, 16) = 6.889, *P* = 0.0184	F (1, 16) = 0.003945, *P* = 0.9507
			Treatment: F (1, 16) = 0.4900, *P* = 0.4940	
3K	2-way RM ANOVA	Sidak's multiple comparisons	Stimulus: F (1, 16) = 6.922, *P* = 0.0182	F (1, 16) = 0.3069, *P* = 0.5872
			Treatment: F (1, 16) = 0.3850, *P* = 0.5437	
3L	2-way RM ANOVA	Fisher's LSD	Stimulus: F (1, 15) = 4.636, *P* = 0.0480	F (1, 15) = 0.01604, *P* = 0.9009
			Treatment: F(1, 15) = 0.2471, *P* = 0.6264	
3M	2-way RM ANOVA	Fisher's LSD	Stimulus: F (1, 16) = 4.320, *P* = 0.0541	F (1, 16) = 0.1170, *P* = 0.7368
			Treatment: F (1, 16) = 1.579, *P* = 0.2269

**Table 3:** Chi-squared statistics table for behavioral responses during Looming Shadow experiments between DO34- and vehicle-treated mice

Vehicle vs DO34			
Behavior Response	P_0_	Chi-squared	*p*-value
No Response	0.28	12.6984	0.0004
Freeze	0.03	3.0928	0.0786
Dart	0.17	1.7718	0.1832
Freeze to Dart	0.12	1.5152	0.2184
Timed Out	0.4	32.6667	1.09E-08

**Table 4:** Statistical Analyses of SCS Habituation with DO34 and Vehicle treatments

Figure	Statistical Test	*p*-value
S1B	Paired t-test	0.445
S1D	Paired t-test	0.6636

**Table 5:** Statistical Analyses of SCS Conditioning with JZL-184, PF-3845, and Vehicle treatments

Figure	Statistical Test	Post-hoc Analysis	Main Effects	Interactions
S3A	2-way RM ANOVA	Dunnet's multiple comparisons	Stimulus: F (2.664, 31.96) = 9.644, *P* = 0.0002	F (80, 480) = 0.7208, *P* = 0.9641
			Treatment: F (2, 12) = 0.05652, P=0.9453	
S3B	2-way RM ANOVA	Sidak's multiple comparisons	Stimulus: F (1, 16) = 73.28, *P* < 0.0001	F (2, 17) = 0.01132, *P* = 0.9887
			Treatment: F (1, 16) = 0.001781, P=0.9669	
S3C	2-way RM ANOVA	Dunnet's multiple comparisons	Stimulus: F (5.909, 70.90) = 183.0, *P* < 0.0001	F (78, 468) = 6.287, *P* < 0.0001
			Treatment: F (2, 12) = 0.5522, P=0.5896	
S3D	2-way RM ANOVA	Sidak's multiple comparisons	Stimulus: F (1, 17) = 4.375, P = 0.0518	F (2, 17) = 0.5506, *P* = 0.5866
			Treatment: F (2, 17) = 3.632, P=0.0486	
S3E	2-way RM ANOVA	Dunnet's multiple comparisons	Stimulus: F (7.207, 86.49) = 42.52, *P* < 0.0001	F (80, 480) = 2.596, *P* < 0.0001
			Treatment: F (2, 12) = 7.732, P=0.0070	
S3F	2-way RM ANOVA	Sidak's multiple comparisons	Stimulus: F (1, 17) = 76.51, *P* < 0.0001	F (2, 17) = 2.263, *P* = 0.1344
			Treatment: F (2, 17) = 0.1577, P=0.8553	
S3G	2-way RM ANOVA	Dunnet's multiple comparisons	Stimulus: F (6.817, 81.80) = 40.75, *P* < 0.0001	F (78, 468) = 5.387, *P* < 0.0001
			Treatment: F (2, 12) = 10.95, P=0.0020	
S3H	2-way RM ANOVA	Sidak's multiple comparisons	Stimulus: F (1, 17) = 84.88, *P* < 0.0001	F (2, 17) = 0.1775, *P* = 0.8389
			Treatment: F (2, 17) = 1.781, P=0.1985

**Table 6:** Statistical Analyses of SCS Extinction with JZL-184, PF-3845, and Vehicle treatments

Figure	Statistical Test	Post-hoc Analysis	Main Effects	Interactions
S5A	2-way RM ANOVA	Dunnet's multiple comparisons	Stimulus: F (8.243, 370.9) = 37.38, *P* < 0.0001	F (80, 1800) = 1.395, *P* = 0.0133
			Treatment: F (2, 45) = 15.93, P<0.0001	
S5B	2-way RM ANOVA	Sidak's multiple comparisons	Stimulus: F (1, 17) = 227.5, *P* < 0.0001	F (2, 17) = 0.03739, *P* = 0.9634
			Treatment: F (2, 17) = 3.250, P=0.0638	
S5C	2-way RM ANOVA	Dunnet's multiple comparisons	Stimulus: F (11.15, 501.7) = 26.92, *P* < 0.0001	F (78, 1755) = 1.604, *P* = 0.0008
			Treatment: F (2, 45) = 1.782, P=0.1799	
S5D	2-way RM ANOVA	Sidak's multiple comparisons	Stimulus: F (1, 17) = 62.86, *P* < 0.0001	F (2, 17) = 0.4493, *P* = 0.6455
			Treatment: F (2, 17) = 0.1120, P=0.8947	
S5E	2-way RM ANOVA	Dunnet's multiple comparisons	Stimulus: F (1, 17) = 214.3, *P* < 0.0001	F (2, 17) = 0.6706, *P* = 0.5244
			Treatment: F (2, 17) = 1.204, P=0.3243	
S5F	2-way RM ANOVA	Sidak's multiple comparisons	Stimulus: F (1, 17) = 51.20, *P* < 0.0001	F (2, 17) = 1.533, *P* = 0.2443
			Treatment: F (2, 17) = 0.1348, P=0.8748	
S5G	2-way RM ANOVA	Dunnet's multiple comparisons	Stimulus: F (1, 17) = 31.70, *P* < 0.0001	F (2, 17) = 0.08543, *P* = 0.9185
			Treatment: F (2, 17) = 4.765, P=0.0228	
S5H	2-way RM ANOVA	Sidak's multiple comparisons	Stimulus: F (1, 17) = 22.58, *P* = 0.0002	F (2, 17) = 0.2607, *P* = 0.7736
			Treatment: F (2, 17) = 0.1223, P=0.8857

**Table 7:** Chi-squared statistics table for behavioral responses to Looming Shadow with JZL-184, PF-3845, and Vehicle treatments

	Vehicle vs JZL-184			Vehicle vs PF-3845		
Behavior Response	P_0_	Chi-squared	*p*-value	P_0_	Chi-squared	*p*-value
No Response	0.29	1.3784	0.2404	0.40	12.8864	**0.0003**
Freeze	0.29	3.4364	0.0638	0.32	0.8367	0.3603
Dart	0.33	0.0191	0.8901	0.23	6.3813	**0.0115**
Freeze to Dart	0.10	0.6792	0.4099	0.05	1.6055	0.2051
Timed Out	N/A	N/A	N/A	N/A	N/A	N/A

## Results

### Pharmacological inhibition of 2-AG synthesis affects both passive and active defensive responses in the SCS paradigm

**Fig. 1:**
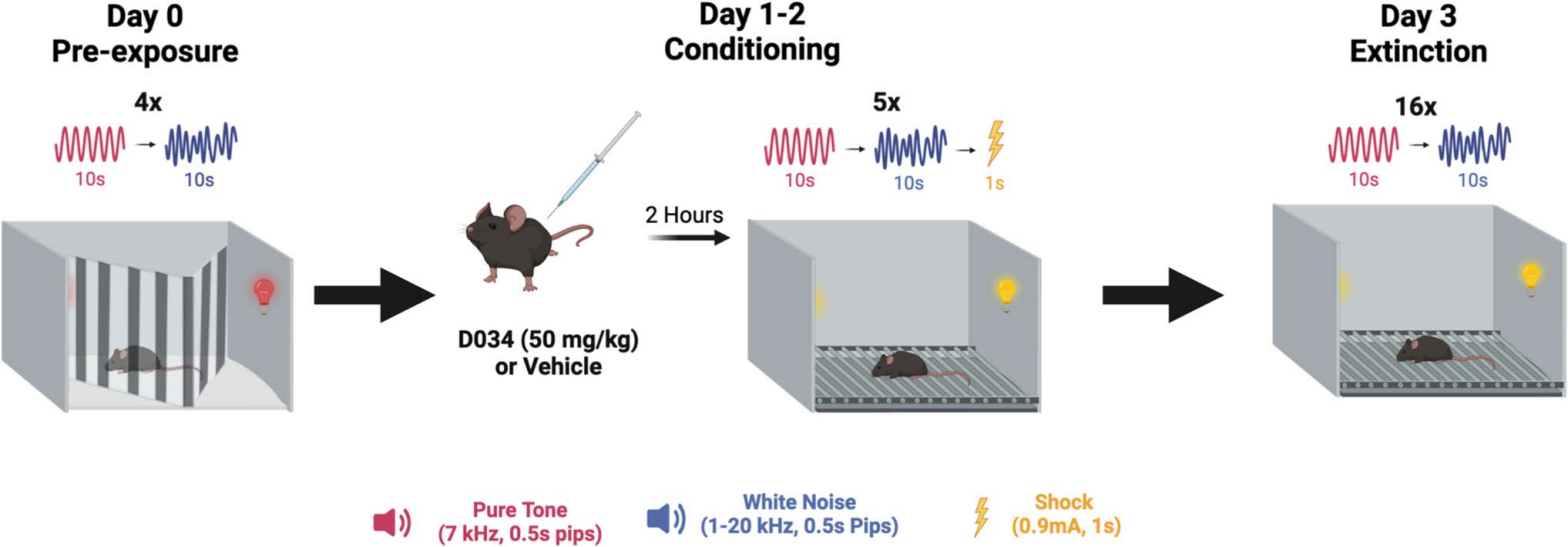
Schematic of Serial Compound Stimulus Paradigm (SCS). The experimental timeline includes three phases: Day 0 (Pre-exposure), Days 1–2 (Conditioning), and Day 3 (Extinction). On Day 0, mice are exposed to alternating presentations of a pure tone (7 kHz, 0.5-s pips) and white noise (1–20 kHz, 0.5-s pips) for 10 s each, repeated four times with pseudorandom intertrial intervals. On Days 1–2, mice receive an intraperitoneal injection of DO34 (50 mg/kg) or vehicle 2 h prior to conditioning. During conditioning, the auditory stimuli are presented in the same sequence, with each trial ending in a 1-s foot shock (0.9 mA). The sequence is repeated five times. On Day 3, mice undergo extinction trials consisting of 16 repetitions of the auditory stimuli without foot shocks

**Fig. 2:**
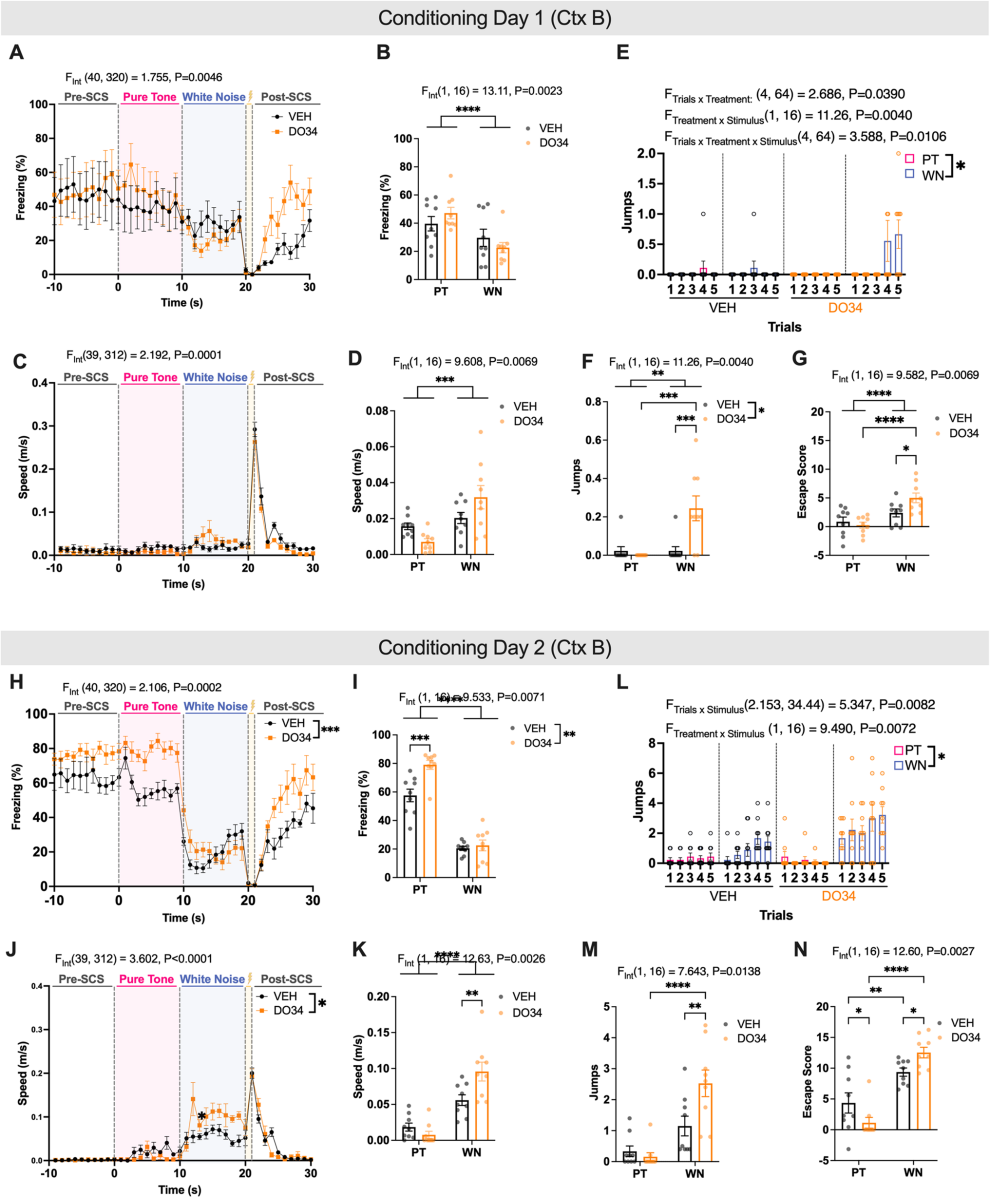
Attenuating 2-AG on conditioning days heightens freezing and flight behavior. **A** Schematic of serial compound stimulus (SCS) paradigm (**B**-**H**) Data are from conditioning day 1. (I-O) Data are from conditioning day 2. **B**, **I** Average freezing trace of vehicle- (VEH, n = 4 males, 5 females) and DO34-(n = 4 males, 5 females) treated mice across SCS presentations. **C**, **J**) Average freezing during PT and WN by treatment group. **D**, **K** Average speed trace of VEH and DO34 treated mice across SCS presentations. **E**, **L** Average speed during PT and WN by treatment group. **F**, **M** Number of jumps during PT and WN per trial. **G**, **N** Average number of jumps across all trials during PT and WN by treatment group. **H**, **O** Average escape scores across all trials during PT and WN by treatment group. All values are presented as mean ± SEM. *p < 0.05, **p < 0.01, ***p < 0.001, ****p < 0.0001

SCS paradigm consisted of a pre-exposure (habituation) day followed by two days of conditioning and one day of extinction (Fig. 1). SCS presentation on the pre-exposure day did not induce freezing or flight responses (Supplemental Fig. 1, Table 4). To test the effect of 2-AG signaling on active flight responses (fleeing and jumping) versus passive (freezing) responses to the SCS, we pharmacologically inhibited 2-AG synthesis with DO34 (50 mg/kg) 2 h before both days of conditioning (Fig. 2A, Table 1). Across both days, DO34-treated mice exhibited higher freezing during PT and lower freezing during WN compared to vehicle-treated mice [stimulus × treatment interaction: Day 1, F (39, 312) = 2.192, *P* = 0.0001 (Fig. 2C); Day 2, F (39, 312) = 3.602, *P* < 0.0001 (Fig. 2J), Table 1]. Conversely, speed was reduced during PT and increased during WN for DO34-treated mice [stimulus × treatment interaction: Day 1, F (1, 16) = 11.26, *P* = 0.0040 (Fig. 2E); F (1, 16) = 9.490, *P* = 0.0072 (Fig. 2L), Table 1].

We also analyzed jumping behavior as a specific subset of flight responses between vehicle and DO34-treated mice. Jumping behavior was significantly influenced by treatment [main effect of treatment: Day 1, F(1,16) = 7.902, *P* = 0.0125; Day 2, F (1, 16) = 4.167, *P* = 0.0581 (Fig. 2F, M), Table 1] and interacted with trial and stimulus context [Day 1, treatment × trials × stimulus interaction: F(4,64) = 3.588, *P* = 0.0106] (Fig. 2E, Table 1). DO34-treated mice exhibited more jumps during WN compared to vehicle-treated controls (Fig. 2G, Table 1). To further quantify flight responses, escape scores were calculated using speed and jump data (Fadok et al. 2017; Hersman et al. 2020; Borkar et al. 2020; Le et al. 2024); see *Materials and Methods*). DO34-treated mice had significantly higher escape scores than vehicle-treated mice during WN on both days [Day 1, *P* = 0.0164; Day 2, *P* = 0.0462], while vehicle-treated mice displayed higher escape scores during PT on Day 2 (*P* = 0.0429) (Fig. 2G, N, Table 1).

Next, we wanted to determine whether enhancing eCB levels could reduce the magnitude of defensive responses to SCS. Thus, mice were treated with JZL-184 (inhibitor of the 2-AG degradation enzyme, MAGL), PF-3845 (inhibitor of the AEA degradation enzyme, FAAH), or vehicle. Freezing differences between PT and WN were observed across all groups, but no significant treatment effects were detected (Supplemental Fig. 3, Table 5). On Day 1, JZL-184-treated mice displayed increased speed during WN [main effect of treatment, *P* < 0.05], but this effect disappeared by Day 2, with stimulus type driving speed differences across groups (Supplemental Fig. 3, Table 5).

**Fig. 3:**
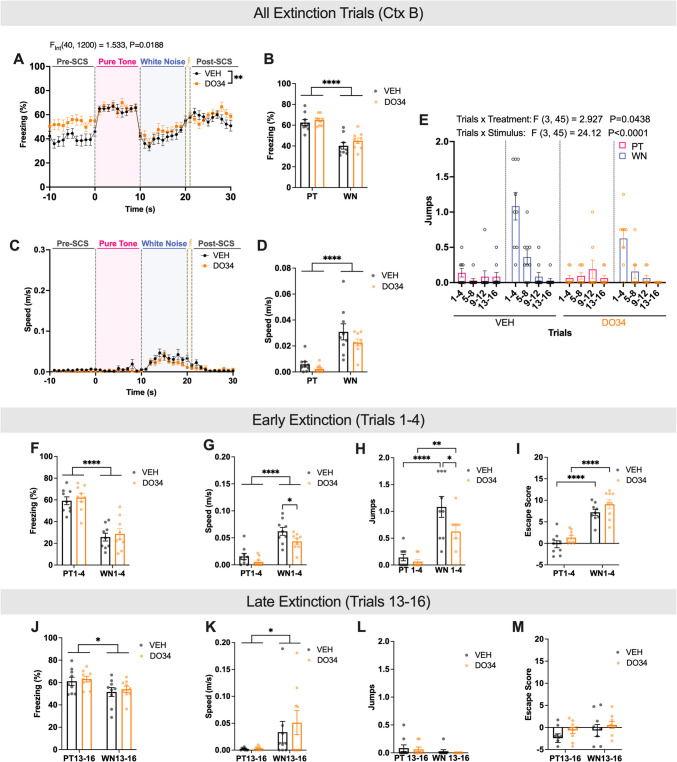
Attenuating 2-AG during fear acquisition has lasting effects on escape behavior during early extinction. **A**-**E** Data are averages from all trials on extinction day. **A** Average freezing trace of vehicle- (VEH, n = 4 males, 5 females) and DO34-(n = 4 males, 5 females) treated mice across all 16 SCS presentations. **B** Average freezing during PT and WN by treatment group. **C** Average speed trace of VEH and DO34 treated mice across SCS presentations. **D** Average speed during PT and WN by treatment group. **E** Average number of jumps during PT and WN per every four trials. **F**-**I** Data are averages from early extinction trials (Trials 1–4). **J**-**M** Data are averages from late extinction trials (Trials 13–16). **F**, **J** Average freezing during PT and WN by treatment group. **G**, **K** Average speed during PT and WN. **H**, **L** Average number of jumps during PT and WN by treatment group. (I, M) Average escape scores during PT and WN by treatment group. All values are presented as mean ± SEM. *p < 0.05, **p < 0.01, ***p < 0.001, ****p < 0.0001

To assess the lasting effects of DAGL inhibition during conditioning on extinction of SCS-driven behavior, mice underwent a single extinction session in the same conditioning context drug-free, receiving 16 SCS presentations without shock. Extinction learning showed no significant interaction between stimulus type and treatment for freezing, speed, or jumps across all trials (Fig. 3A-E, Table 2). To evaluate potential differences in extinction rates, trials were divided into early (trials 1–4) and late (trials 13–16) stages. During early extinction, previously DO34-treated mice exhibited slower speeds [Sidak’s post-hoc, *P* = 0.0433 (Fig. 3G, Table 2)] and fewer jumps during WN [Sidak’s post-hoc, *P* = 0.0162 (Fig. 3H, Table 2)]. DO34-treated mice also had marginally higher escape scores during WN, reflecting divergence from baseline active responses rather than overall activity levels. Despite persistent differences in freezing and speed between PT and WN, no treatment effects were detected.

The impact of enhanced eCB signaling during acquisition on extinction was also assessed. Similar to 2-AG attenuation, a main effect of stimulus type on freezing and speed was observed during extinction. However, treatment effects were absent during early extinction and when averaging all trials. Notably, a main effect of treatment on speed emerged during late extinction trials (F (2, 17) = 4.765, *P* = 0.0228) (Supplemental Fig. 5G, Table 6).

### Pharmacological inhibition of 2-AG synthesis biases towards passive defensive behaviors during innate threat

**Fig. 4:**
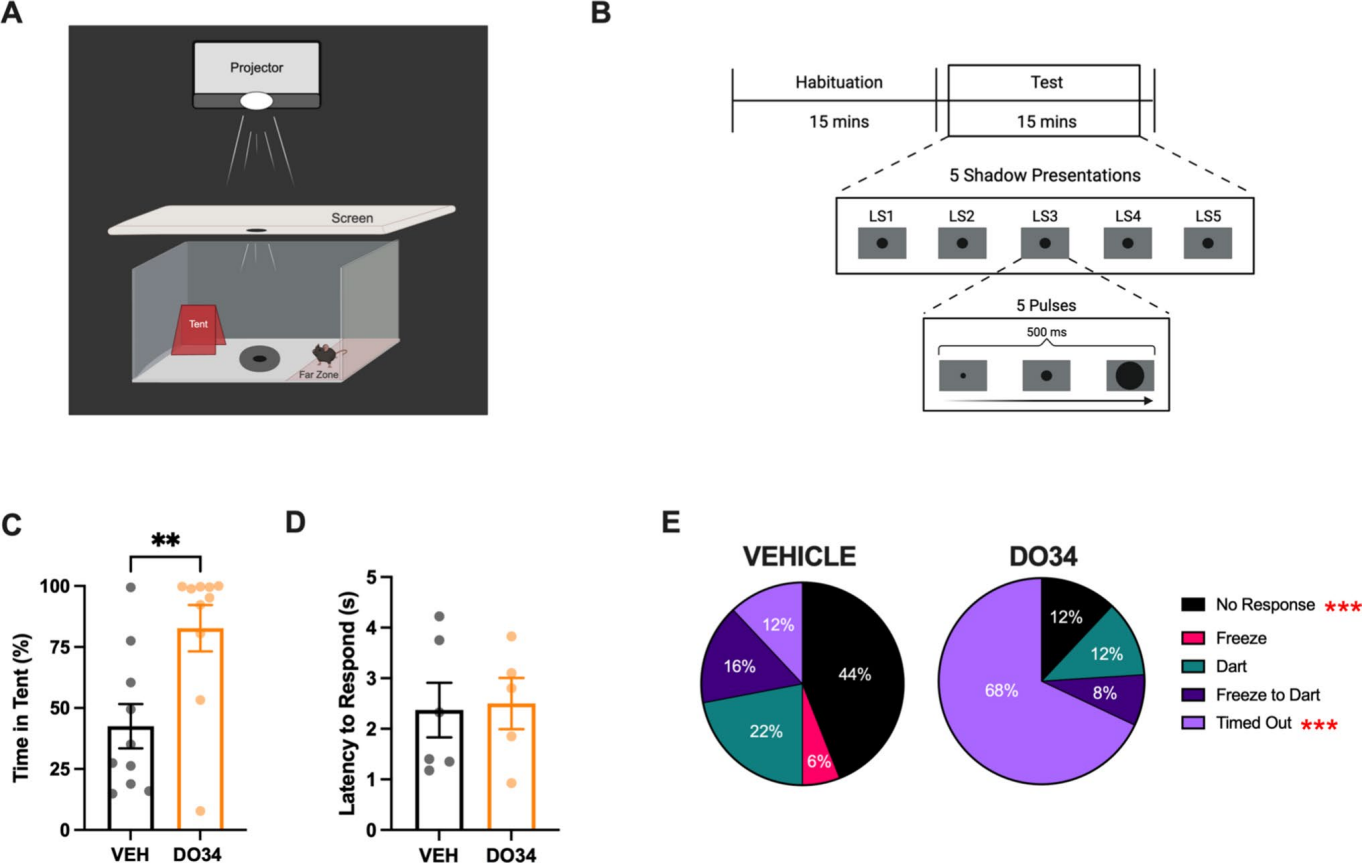
Attenuating 2-AG biases passive state responses to innate fear. **A** Looming shadow arena (**B**) Behavioral paradigm (**C**) Percent time spent in tent during test stage. **D** Average latency to respond to shadow presentation. **E** Average percentage of times a behavior was displayed in response to shadow by all mice in each treatment group (VEH: n = 5 males, 5 females; DO34: n = 5 males, 5 females). Differences between treatment groups were determined by chi-squared test. All error bars represented as ± SEM. *p < 0.05, **p < 0.01, ***p < 0.001, ****p < 0.0001

To test whether 2-AG signaling affects defensive response strategies in response to innate threat, mice were treated with vehicle or DO34 and underwent a looming shadow protocol that elicits both active and passive defensive responses (Daviu et al. 2020a, 2020b). In an open arena with a tent “safe zone”, mice were exposed to up to five presentations of an overhead, growing circular shadow simulating an aerial predator (Fig. 4A-B, see *Materials and Methods*). DO34-treated mice spent significantly more time in the tent compared to vehicle-treated controls during the testing phase (Fig. 4C), though there were no differences in response latency to the shadow presentations (Fig. 4D). Notably, vehicle-treated mice exhibited more instances of no response to the shadows, whereas DO34-treated mice timed out significantly more often (Fig. 4E, Table 3, Supplemental Fig. 6). These findings suggest that 2-AG deficiency is associated with enhanced passive defensive responses during repeated innate threat presentation.

Next, we examined whether augmenting eCB signaling affected defensive responses to the looming shadow. Similar to the DO34-treated mice, there were no differences in the latency to respond to shadows between vehicle- JZL-184-, and PF-3845-treated groups (Supplemental Fig. 7A, Table 8 ). Additionally, there were no differences in the amount of time spent in the tent between the three treatment groups (Supplemental Fig. 7B, Table 8). However, comparisons between vehicle versus PF-3845 revealed a significantly lower proportion of'No Response'in the PF-3845 group (χ^2^ = 12.89, *p* = 0.0003, Table 7) and a significantly higher proportion of'Dart'responses (χ^2^ = 6.38, *p* = 0.0115, Table 7). No other categories differed significantly between these groups. In contrast, vehicle versus JZL-184 comparisons revealed no significant differences were found across individual response categories (all *p* > 0.05, Table 7). These findings suggest that increased AEA levels may promote active state defense responses to innate threats.

**Table 8:** Statistical analyses of Looming Shadow responses with JZL-184, PF-3845, and Vehicle treatments

Figure	Statistical Test	*p*-value
S7A	Kruskal-Wallis test	0.2566
S7B	Kruskal-Wallis test	0.6803

## Discussion

The appropriate selection of behavioral responses in dangerous situations is vital for survival and is influenced by the perceived proximity and intensity of threats. Various studies have investigated the biological underpinnings surrounding the four most common responses to a perceived threat: fight, flight, freeze, and fawn (Zingela et al. 2022). The mechanisms underlying shifts between different response states, however, remain incompletely understood. The eCB has emerged as a key modulator of threat appraisal and defensive behavior selection (Maldonado et al. 2020). The present study extends these findings by delineating the role of the eCB system in shaping passive-to-active behavioral transitions in response to both learned and innate aversive stimuli.

To discern the role of 2-AG in regulating passive (freezing) and active (jumping, fleeing) defensive behaviors, we first employed the SCS paradigm. On day 1 of conditioning, pharmacological reduction of 2-AG via DO34 treatment led to an increased number of jumps during the WN component compared to vehicle-treated mice. Interestingly, on Day 2 we observed that DO34-treatment led to heightened passive responses to the pure tone *and* increased active responses to white noise. It is important to contextualize these findings within the overall freezing patterns across conditioning. On Day 1, average pre-SCS freezing values were elevated (~ 40%) for both treatment groups and freezing during the pure tone and white noise periods did not significantly exceed this baseline. However, freezing levels during trial 1 were comparable to those on Day 0 (Supplemental Fig. 2), suggesting that initial elevated freezing reflects a generalized fear response to the first tone-shock pairing rather than a drug-induced locomotor impairment. This interpretation is further supported by the similar pre-SCS freezing levels between DO34 and vehicle groups, and prior evidence that DO34 does not impair general locomotion (Ramos-Medina et al. 2024). While cue-specific freezing was limited on Day 1, clear cue discrimination emerged by Day 2 and during extinction, indicating intact associative learning. These findings suggest that 2-AG depletion may facilitate fear memory consolidation, thereby amplifying defensive responses during subsequent SCS exposure.

Extinction of conditioned fear responses is also essential for adaptive behavior, enabling reductions in defensive responses when threat is no longer present. Impaired extinction is a hallmark of stress-related disorders, including PTSD (Norrholm et al. 2011). Prior work has demonstrated that diminished eCB signaling can impair fear extinction (Marsicano et al. 2002; Hill et al. 2018; Cavener et al. 2018; Ramos-Medina et al. 2024), whereas enhancement of eCB signaling is linked to facilitation of fear extinction (Chhatwal et al. 2005, 2009; Bitencourt et al. 2008; Gunduz-Cinar et al. 2013, 2023). Interestingly, we found that during early extinction (trials 1–4), previously DO34-treated mice showed significantly attenuated active fear responses (Fig. 3G, H). However, their calculated escape scores were higher than controls, which appears to be a limitation of the metric itself: if a mouse exhibited complete freezing during the pre-SCS period, the resulting score was artificially inflated due to a near-zero denominator (Supplemental Fig. 6). These results suggest that 2-AG may promote fear extinction by preferentially dampening active defensive responses. The neural underpinnings of these effects may involve circuit-specific modulation, as two different cell types in the central amygdala – somatostatin and corticotropin-releasing factor neurons– initiate passive freezing behavior and mediate conditioned flight responses, respectively (Fadok et al. 2017). Thus, we conclude that 2-AG depletion during conditioning promotes the subsequent extinction of active defensive responses to SCS presentation, however the effects of 2-AG depletion during extinction training itself were not evaluated.

In contrast, global enhancement of 2-AG levels via MAGL inhibition (JZL-184) did not have significant effects on fear acquisition or extinction. These findings partially align with a previous work from our lab in which JZL-184 pre-treatment had no impact on cued-conditioning fear learning (Hartley et al. 2016). However, while the former study saw impairment in fear extinction, we did not replicate that effect with our SCS protocol. This discrepancy may stem from the timings of the JZL-184 administration. In the present study, our findings inform us of the effects of varying eCB tone during fear acquisition on extinction learning, as drug treatments only occurred on conditioning days. Meanwhile, our prior work examined the effects of enhanced 2-AG levels during extinction learning and, thus, drug treatments were delivered one hour prior to extinction sessions (Hartley et al. 2016).

Similarly, augmenting AEA levels via FAAH inhibition (PF-3845) did not alter fear learning or extinction. One possible explanation is that naturally occurring AEA signaling may be maximally engaged during conditioning, and further elevations in AEA content cannot affect defensive response generation. This is supported by studies that show increased AEA levels in the medial prefrontal cortex, amygdala, hippocampus, and periaqueductal gray following footshock stress (Hohmann et al. 2005; Morena et al. 2014). However, some studies have demonstrated contrasting results with reductions in AEA content globally (Bluett et al. 2014) and in the amygdala (Vecchiarelli et al. 2022) after footshock stress. Thus, these findings highlight the complexity of AEA signaling dynamics in response to stress, suggesting that its effects on defensive behavior may be region-specific, temporally regulated, and highly sensitive to experimental context.

Taken together, these data indicate that 2-AG deficiency is associated with progressively enhanced magnitude of passive and active responses to SCS across conditioning days, thereby amplifying both defensive response strategies to SCS presentation. However, analysis of escape scores suggests DO34 treatment may bias behavior toward active coping as threat imminence increases, driving a transition from passive to active responses. These data point to an important role for endogenous 2-AG in constraining both active and passive defensive responses during escalating threat imminence.

Adaptive responses to predator-like threats generally fall into two primary categories: (1) avoiding detection through behaviors like freezing and (2) avoiding capture through actions such as fleeing or fighting (Yilmaz and Meister 2013; Shang et al. 2018; Salay et al. 2018). In this study, we used a looming shadow paradigm to elicit these behaviors and examine how eCBs regulate the repertoire and shift of behaviors to a perceived imminent threat. Consistent with previous reports, we observed that the predominant defense responses to looming shadow stimuli were flight/escape behaviors, including darting and transitions from freezing to darting (Fig. 4D) (Yilmaz and Meister 2013; Daviu et al. 2020b). Our results showed that pharmacological inhibition of 2-AG synthesis led to animals spending significantly more time in the tent, with no changes in latency to respond to the shadows. Additionally, DO34-treated mice timed-out significantly more than vehicle-treated mice, suggesting exaggerated passive defensive responses at the expense of exploratory behavior during the inter-trial interval when no threat was present. While we did not analyze behavior as a function of time across the looming stimulus itself, doing so in future work could reveal whether 2-AG dynamically modulates the shift from passive to active responses as the perceived threat escalates. This would offer further insight into how 2-AG regulates adaptive defense strategies in a threat-imminence-dependent manner. Taken together, endogenous 2-AG may be important for terminating passive defensive responses in the absence of threat and promoting optimal exploratory behavior in the absence of threat.

Importantly, these effects appear to be specific to reductions in 2-AG, as enhancing 2-AG levels via the MAGL inhibitor JZL-184 had no significant effect on shadow-evoked behaviors, latency to respond, or time spent in the tent. In contrast, augmenting AEA levels by blocking its degradation promoted more passive behavioral responses, although latency and time in tent remained unaffected. Our data suggest that 2-AG signaling may be necessary for adaptive behavioral transitions in the absence of threat, while AEA may serve to modulate coping style toward more passive responding without altering threat detection or appraisal. These results align with previous work in which mice showed reduced flight behaviors to an approaching robo-beetle after enhancement of AEA levels (Heinz et al. 2017). Similarly, we observed fewer darts and decreased reactivity to shadow stimuli, indicative of a shift toward more passive coping strategies. However, while they also reported that enhanced 2-AG signaling increased flight responses (Heinz et al. 2017), which we did not observe. Notably, their study lacked a designated ‘safe zone’ for the mice to retreat to, which may have influenced the expression of escape responses. This difference in task design may explain the divergent behavioral outcomes and suggests that environmental context can shape how 2-AG modulates threat responses.

One alternative interpretation is that eCBs induce alterations in visual perception that affect defense responses. While it is well-established that eCBs are known to play important roles in visual system development (Bouchard et al. 2016), less is known about the acute effects of 2-AG depletion on visual processing. However, a recent study examined how different cannabinoids affect mouse visual acuity. They report that pharmacological augmentation and attenuation of 2-AG decreased and increased visual acuity, respectively, while augmenting AEA did not affect acuity (Cécyre et al. 2020). This aligns with our finding that DO34 does not impair threat detection, as treated mice displayed normal latencies to respond to the looming stimulus, suggesting intact visual processing. Future studies directly assessing visual function during acute 2-AG depletion will be necessary to fully rule out this possibility.

Beyond behavioral outcomes, future studies should also dissect the neural circuitry by which 2-AG signaling shapes defensive state transitions. Selective targeting of eCB machinery using promising tools such as the DAGLα^f/f^ (Winters et al. 2021) or CB1^f/f^ (Marcus et al. 2020; Kondev et al. 2023b) mouse lines may provide insight into how local eCB tone modulates defensive behavior. Notably, recent work has demonstrated that the visual cortex can instruct the suppression of innate defensive responses through a top-down pathway to the ventrolateral geniculate nucleus (vLGN), with this learning-induced plasticity requiring eCB-mediated long-term suppression of inhibitory synapses onto vLGN neurons (Mederos et al. 2025). Such findings reinforce the idea that eCB signaling is critical for adaptive behavioral plasticity in response to complex and evolving threat environments.

In summary, our study demonstrates that 2-AG signaling plays a critical role in regulating behavioral state transitions across both learned and innate fear responses. During learned fear paradigms, such as the SCS, endogenous 2-AG appears to constrain the magnitude of both passive and active defense strategies, potentially promoting adaptive responses to escalating threat imminence. Similarly, in innate fear paradigms like the looming shadow test, endogenous 2-AG appears to promote exploratory behavior between threat presentations and thus may serve an adaptive role to maximize exploratory/foraging in the absence of threat. These findings highlight the context-dependent role of the endocannabinoid system in facilitating appropriate behavioral selection during perceived threats. By revealing how 2-AG shapes the repertoire and transition of defensive states, this study underscores the broader impact of the eCB system on adaptive responses to fear and stress. Elucidating these mechanisms may ultimately inform novel therapeutic strategies for fear dysregulation in stress-related psychiatric disorders.

## Supplementary Information

Below is the link to the electronic supplementary material. 

ESM1 (PDF 6.83 MB)

## Data Availability

N/A.
