# Supplementary material for: Endocannabinoid modulation of defensive state transitions to innate and learned threat
Source: Psychopharmacology (Berl). 2025 May 24;243(2):325–38. doi: 10.1007/s00213-025-06812-z (PMC12904945; doi:10.1007/s00213-025-06812-z)
Supplement: Supplementary file 1 — (PDF 6.83 MB) [file 213_2025_6812_MOESM1_ESM.pdf]

Psychopharmacology

Endocannabinoid modulation of defensive state transitions to innate and learned threat

--Manuscript Draft--

**Manuscript Number:**	PSPH-D-25-00013R1
**Full Title:**	Endocannabinoid modulation of defensive state transitions to innate and learned threat
**Article Type:**	Original Investigation
**Section/Category:**	Preclinical Psychopharmacology: Behavioral and Neural
**Funding Information:**	NIMH (MH107435) — Professor Sachin Patel
**Abstract:**	A hallmark of many psychiatric disorders is maladaptive and heightened fear responses to non-threatening stimuli. Adaptive defensive responses to threats involve transitions between passive behaviors, such as freezing, and active escape strategies, such as darting or fleeing. The endocannabinoid (eCB) system, particularly 2-arachidonoylglycerol (2-AG), plays a crucial role in modulating fear and stress responses. However, the extent to which 2-AG influences defensive behavioral state transitions to fear responses remains unclear. To address this, we investigated the role of 2-AG in shaping defensive behaviors to learned and innate threats using pharmacological manipulations in both the serial compound stimulus (SCS) and the looming shadow paradigm. During SCS, inhibition of 2-AG synthesis enhanced freezing to early cues and promoted active responses during cues associated with heightened threat imminence. In the looming shadow paradigm, 2-AG depletion biased defensive behavior toward freezing and increased time spent in a safe zone, suggesting a shift toward passive responses. These findings demonstrate that 2-AG signaling critically regulates the balance and transitions between passive and active defensive strategies in both learned and innate fear contexts. Thus, 2-AG plays a key role in the scaling of defensive response transitions and the promotion of active defensive responses to threats.
**Corresponding Author:**	Sachin Patel<br>Northwestern University Feinberg School of Medicine<br>UNITED STATES OF AMERICA
**Corresponding Author Secondary Information:**	
**Corresponding Author's Institution:**	Northwestern University Feinberg School of Medicine
**Corresponding Author's Secondary Institution:**	
**First Author:**	Niharika Loomba
**First Author Secondary Information:**	
**Order of Authors Secondary Information:**	
**Order of Authors:**	Niharika Loomba
	Anyu Cao
	Senna Charles
	Isaac Kandil
	Michelle Kwon
	Sachin Patel
**Author Comments:**	

Powered by Editorial Manager® and ProduXion Manager® from Aries Systems Corporation

Click here to access/download;Author's Response to Reviewers' Comments;Response to Reviewers.docx

We thank the reviewers for the time and attention to this manuscript. We believe the points raised were important and have done our best to address all of the changes suggested by the reviewers. We feel the work has been much improved by their suggestions. Below, please find each concern raised by the reviewer and our response.

Introduction:

This section is written well.

1-1. Please include on the first page (line 48) the following citations: Kathuria et al 2002 and Gunduz-Cinar 2013

We thank the reviewer for providing additional citations. We have included the suggested citations in the main text and bibliography.

1-2. Correct the formatting of some cited references here and in other sections of the manuscript (eg. Correct "Daviu, Füzesi et al 2020" to "Daviu et al 2020")

We thank the reviewer for their feedback on proper formatting of citations. We have now revised the citation to "Daviu et al., 2020a" and "Daviu et al., 2020b" as one is a *Bio-protocol* publication, and the other is a *Nature Neuroscience* publication.

Material and Methods:

This section needs to be revised.

2.1. The serial compound stimulus paradigm is mentioned differently in figure 1 and methods section, make both the same.

Thank you for pointing this out. We have corrected this error so the stimulus is mentioned the same throughout the figures and the text.

2.2. Day 0 (figure 1) is mentioned as day 1 in the text, please correct.

Thank you for pointing this out. We have corrected all references to Pre-exposure day as Day 0 in the figures and the main text.

2.3. Escape Score Calculations section, line 48 correct typos "dring to during" and "1os to 10s"

We thank the reviewer for their attention to detail. We have corrected the typos and done our best to ensure there are no others.

2.4. Does the vehicle of the ip injections for JZL184 and PF-3854 include only DMSO (meaning 100%?). It's not acceptable to give just DMSO solution. Authors should apply 1:1:18 solution similar to DO34 preparation.

We thank the reviewer for this important point. We confirm that the vehicle for JZL-184 and PF-3854 was 100% DMSO. This formulation has been consistently used by our lab in numerous studies and is supported by previously published work (Kondev et al., 2022; Morgan et al., 2022; Kondev et al., 2023). While we acknowledge that alternative vehicles such as 1:1:18 are also used, we have found 100% DMSO to be effective for solubilizing these compounds and well-tolerated in our experimental paradigm. To avoid confusion, we have now clarified the vehicle composition in the Methods section and added citations to our previous work using this approach.

Results and Discussion

3.1. Why does the preSCS freezing values on day 1 conditioning are high for both treatment groups? It's about 40% which complicates the interpretation of the rest of the data presented. (Are

the freezing responses for pure tone and white noise significantly different than the pre-SCS freezing responses?)
Same for extinction trials too. The baseline value before the first pure tone on habituation day seems lower, this is in the absence of drug treatment. If DO34 doesn't induce locomotor problems, why would the baseline (pre SCS periods) have high freezing values both for vehicle and DO34?

We appreciate the reviewer's observation. It is correct that average pre-SCS freezing values across the 5 trials are elevated (~40%) on Day 1 for both treatment groups, and in our dataset, freezing during the pure tone and white noise periods is not significantly higher than the pre-SCS baseline. This pattern is also observed during extinction, where pre-SCS freezing remains elevated.

Below, we have included the average freezing levels for trials 1, 3, and 5 on Conditioning Day 1. Notably, the freezing levels for trial 1 are comparable to those observed during Day 0. Thus, we interpret the elevated freezing levels following trial 1 to be a generalized fear response following the first tone-shock pairings, rather than a drug-induced locomotor impairment. This interpretation is supported by the similarity in pre-SCS freezing levels between vehicle- and DO34-treated animals, as well as prior work showing that DO34 does not alter general locomotor activity (Ramos-Medina et al., 2024). We have now included these figures into the Supplement to improve clarity of the data.

While the lack of significant cue-specific freezing on Day 1 limits interpretation at that time point, we note that robust cue discrimination emerges on Day 2 and during extinction trials, indicating successful associative learning. We have now clarified this in the Results and Discussion.

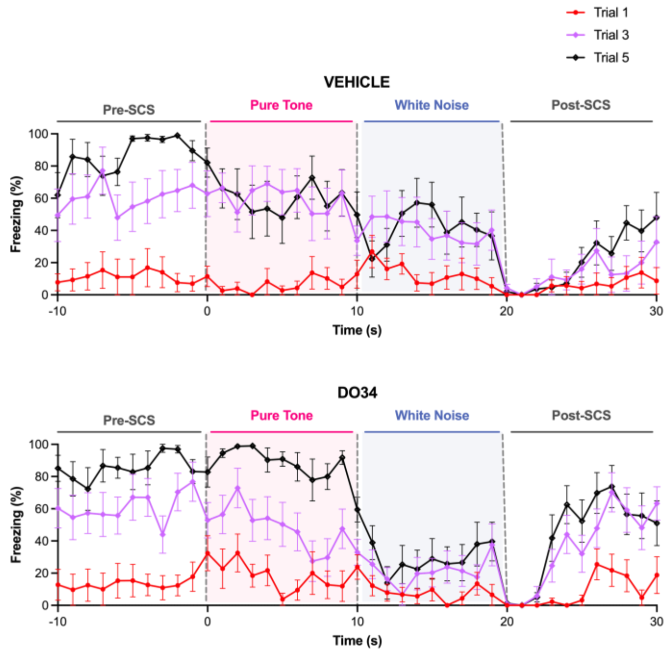

3.2. Not able to find Fig 2O or supplementary figure 2O. Please delete from main text and figure legends. It's unclear what statistical values are referring to that. Similarly Supplementary Figure 2 legend has to be corrected according to the Figures A-H.

Thank you for pointing out this error. We have deleted any references to “Figure 2O” and corrected the figure legend for Supplementary Fig. 2.

3.3. Have the authors injected FAAH inhibitor after the DO34 treatment to compensate the possible decrease in AEA content in their behavior? What about the DAGLa knockout mice?
Thank you for this thoughtful suggestion. We did not perform experiments involving FAAH inhibitor administration following DO34 treatment in the current study, nor did we utilize the DAGL KO mice. However, these are

3.4. Statistical results shown on Figure 2C and 2J doesn't match with what's written in the main text (results section line 29-31) same for the speed data shown on lines 34 and 36 and jump data shown on lines 43-46.
Thank you for pointing out this error. We have edited the main text so that it matches the statistical results in Table 1. We have also added additional references to Table 1 where necessary to improve clarity.

3.5. Is it possible to see the binned data for post SCS values in Fig 2-3?
Certainly. Below, you will see the percent freezing by trial for only the post-SCS time period.

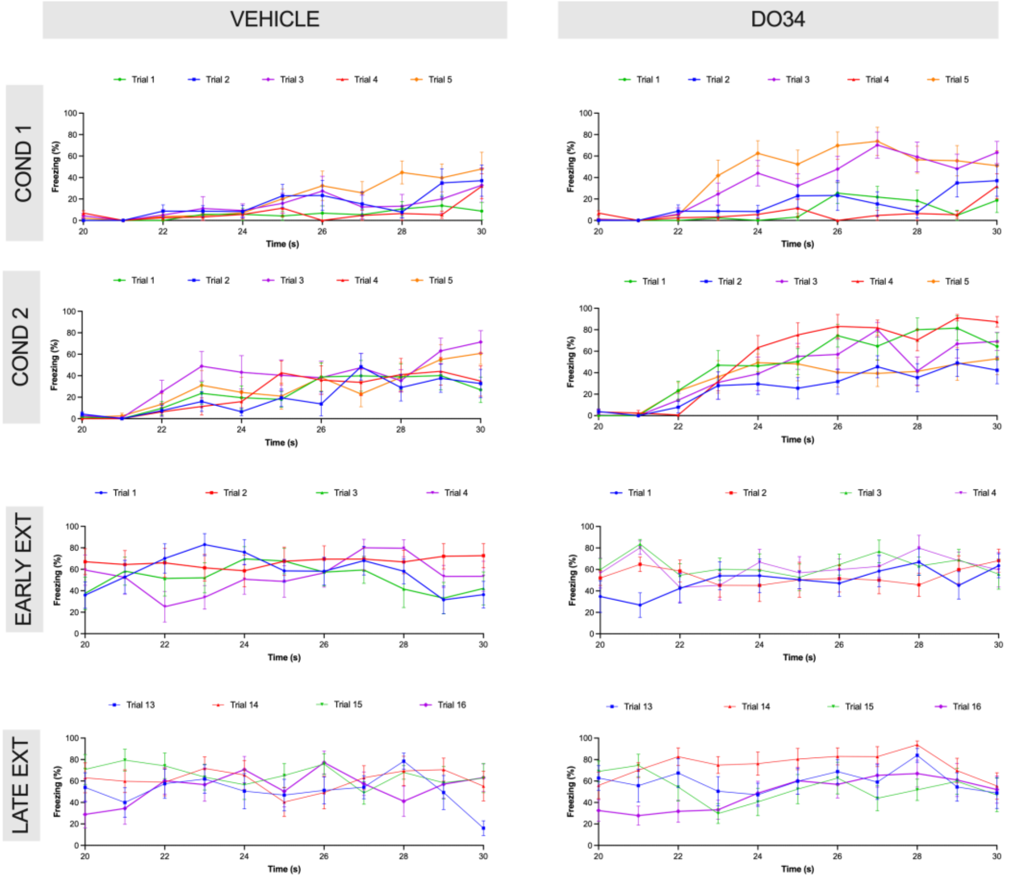

3.6. Have the authors tried JZL184 for looming shadow behavior?
We thank the reviewer for this question. This point was also raised by another reviewer (Reviewer 2, Comment 1), and we have included a detailed response there. Briefly, we did test JZL184 in the looming shadow task and found that it did not significantly alter behavioral responses, including latency to respond, distribution of response types, or time spent in the tent. These results suggest that enhancing 2-AG signaling does not affect innate defensive behavior in this context. We have now clarified these findings in the Results and Discussion sections and added the relevant data to the Supplements.

3.7. Have the authors increased the sessions to an extent where mice learn that the shadows are non-threating? (once this learning occurred would the DO34 treated mice still be spending more time in the tent?) Is there any difference in the displayed behaviors from the first illumination to the last?
Thank you for raising this important question. At this time, we have not extended the test session length beyond 15 minutes as the DO34-treated mice typically stayed in the tent until they were removed from the apparatus. As such, it is hard to say if there are differences in the displayed behaviors from the first shadow to the last, as many DO34-treated mice only experienced a couple of shadow presentations because they did not leave the tent. However, future experiments in which mice are exposed to the shadows on multiple days would certainly provide valuable insight into how 2-AG signaling modulates defensive responses when a stimulus is no longer deemed a threat.

3.8- We know that the endocannabinoids are important in visual system development. Is there any data on acute effects of 2-AG depletion on vision? This could be important in the interpretation of the looming shadow experiments. Please discuss.
Thank you for this suggestion. We have included the following paragraph in the discussion:
"One alternative interpretation is that eCBs induces alterations in visual perception that affect defense responses. While it is well-established that eCBs are known to play important roles in visual system development (Bouchard et al. 2016), less is known about the acute effects of 2-AG depletion on visual processing. However, a recent study examined how different cannabinoids affect mouse visual acuity. Here, they report that pharmacological augmentation and attenuation of 2-AG decreased and increased visual acuity, respectively, while augmenting AEA did not affect acuity (Cécyre et al. 2020). This aligns with our finding that DO34 does not impair threat detection, as treated mice displayed normal latencies to respond to the looming stimulus, suggesting intact visual processing. Future studies directly assessing visual function during acute 2-AG depletion will be necessary to fully rule out this possibility."

3.9. Based on the recent Mederos et al 2024 paper have the authors consider using the available DAGL floxed mice to specifically manipulate the 2-AG content in a circuit specific manner? Please discuss.
We appreciate the reviewer's thoughtful suggestion. Indeed, the use of DAGL floxed mice to achieve cell- or circuit-specific manipulation of 2-AG signaling represents an exciting avenue for future studies. However, such targeted genetic approaches fall beyond the scope of the current

project, which was designed to broadly assess the behavioral consequences of global pharmacological inhibition of 2-AG synthesis. We agree that circuit-specific manipulation will be valuable for future mechanistic studies to further delineate the cell-type and pathway-specific roles of 2-AG in stress-related behaviors and have added this point in the discussion.

"Beyond behavioral outcomes, future studies should also dissect the neural circuitry by which 2-AG signaling shapes defensive state transitions. Selective targeting of eCB machinery using promising tools such as the DAGL□f/f (Schuele et al. 2022) or CB1f/f (Marcus et al. 2020; Kondev et al. 2023b) mouse lines may provide insight into how local eCB tone modulates defensive behavior. Notably, recent work has demonstrated that the visual cortex can instruct the suppression of innate defensive responses through a top-down pathway to the ventrolateral geniculate nucleus (vLGN), with this learning-induced plasticity requiring eCB-mediated long-term suppression of inhibitory synapses onto vLGN neurons (Mederos et al. 2025). Such findings reinforce the idea that eCB signaling is critical for adaptive behavioral plasticity in response to complex and evolving threat environments."

Reviewer #2: The submitted work investigates the role of 2-AG in passive and active defense behaviors by pharmacologically inhibiting 2-AG synthesis and 2-AG degradation prior to two different threat paradigms. In the first paradigm, the authors have shown a detailed breakdown of Passive (freezing) and active (movement speed and jumping) defensive behaviors in response to successive presentations of pure tone, white noise, and foot shock. These measures have outlined a behavioral profile consisting of the transition from passive behaviors to active behaviors as the impending threat approaches in time. Later in conditioning, mice administered DO34 appear to elevate freezing during pure tone and increase escape scores during white noise. This is interpreted as an elevation of the natural fear response in the paradigm, suggesting that DO34 increases fear consolidation. This means that 2-AG is important for constraining both active and passive responses as the threat approaches.
Animals who received DO34 before conditioning also appeared to show increased active behaviors during white noise, suggesting a role for 2-AG in promoting fear extinction particularly for active defense behaviors. MAGL and FAAH inhibition had no effect in this paradigm.

Additionally, the authors investigated the role of 2-AG in a looming threat paradigm. Animals who had blunted 2-AG synthesis exhibited increased time in tent and increased responses to the looming threat when compared to vehicle animals. The authors interpreted this as a role for 2-AG in terminating the passive defensive strategy (hiding) and promoting exploration in the absence of a threat.

The experimental procedures are well designed and executed and the story, while disjointed, is mostly clear and consistent. I only have a few comments that need to be addressed.

1. The addition of the looming threat paradigm feels tacked on at the end and does not really add

much to the story. These results are briefly discussed in tandem in the conclusion paragraph. However, more discussion around how the looming threat paradigm adds to the overarching theme (2-AG regulation of both active and passive defense behaviors depending on the imminence of the threat) is warranted. This could possibly be achieved by looking at behaviors as the shadow grows in size to see if DO34 affects behavior as the threat approaches.
We thank the reviewer for their suggestion. We have added the following to our discussion section with respect to this point:
"While we did not analyze behavior as a function of time across the looming stimulus itself, doing so in future work could reveal whether 2-AG dynamically modulates the shift from passive to active responses as the perceived threat escalates. This would offer further insight into how 2-AG regulates adaptive defense strategies in a threat imminence-dependent manner"

2. While MAGL and FAAH were inhibited in the SCS paradigm, there was no effort to investigate how elevating 2-AG and AEA may influence behaviors in the looming shadow paradigm. You mention multiple studies that have investigated how 2-AG augmentation has significantly influenced behavior during an approaching or present threat, yet you don't attempt to investigate this in the looming threat paradigm. Please address why you did not approach both paradigms with the same pharmacological interventions.
We thank the reviewer for this important point and agree wholeheartedly that the lack of MAGL and FAAH inhibition leaves the story disjointed. We have since conducted these experiments and found that MAGL inhibition via JZL-184 had no significant impact on behavior, while FAAH inhibition via PF-3845 biased responses to shadows as those of active states. Both treatments had no impact on latency to respond to shadows or time spent in the tent. These findings are now included in the main text of the results, Supplemental Figure 8, and Tables 4 and 7.
3. You did not see any effects of MAGL or FAAH inhibition on active or passive defensive behaviors in the SCS paradigm. This was interpreted as a ceiling effect of the eCB system where endocannabinoids are maximally engaged during conditioning. However, multiple reports have noted decreases in AEA following stress, particularly in the amygdala. The effects of fear conditioning (foot shocks) on AEA levels should be further discussed.
Thank you for pointing out this important point. The paragraph below has been added to the discussion section:

"Similarly, augmenting AEA levels via FAAH inhibition (PF-3845) did not alter fear learning or extinction. One possible explanation is that naturally occurring AEA signaling may be maximally engaged during conditioning, and further elevations in AEA content cannot affect defensive response generation. This is supported by studies that show increased AEA levels in the medial prefrontal cortex, amygdala, hippocampus, and periaqueductal gray following footshock stress (Hohmann et al. 2005; Morena et al. 2014). However, some studies have demonstrated contrasting results with reductions in AEA content globally (Bluett et al. 2014) and in the amygdala (Vecchiarelli et al. 2022) after footshock stress. Thus, these findings highlight the complexity of AEA signaling dynamics in response to stress, suggesting that its effects on defensive behavior may be region-specific, temporally regulated, and highly sensitive to experimental context."

Minor:

1. Line 36: "suggesting endogenous eCB signaling" feels redundant. Baseline or naturally occurring would be better

We thank the reviewer for their suggestion to improve the conciseness of the text. We have changed the phrasing to “naturally occurring.”

2. Line 36: "may me maximally" should be "may be maximally"

Thank you for notifying us of this typo. We have edited the text to read “may be maximally."

3. Line 43: "data indicate 2-AG deficiency" should be "data indicate that 2-AG deficiency:

Thank you for the suggestion. We have changed the phrasing to “data indicate that 2-AG deficiency.”

Manuscript

Click here to access/download;Manuscript;Clean copy Manuscript.docx

Endocannabinoid modulation of defensive state transitions to innate and learned threat

Niharika Loomba[1,2†], Anyu Cao[2†‡], Senna Charles[2], Isaac Kandil[2], Michelle Kwon[2], Sachin Patel[2*]

[1]Vanderbilt Brain Institute, Vanderbilt University, Nashville, TN USA, [2]Stephen M. Stahl Centre for Psychiatric Neuroscience, Department of Psychiatry and Behavioral Sciences Northwestern University Feinberg School of Medicine, Chicago IL, USA

[†]Authors contributed equally
[‡] Current affiliation: Neuroscience Graduate Program, University of California, San Francisco, CA, USA

Number of
Words: 4596
Figures: 4 Main Figures, 7 Supplemental Figures
Tables: 7
Boxes: N/A
Movies: N/A
Link to websites: N/A

Keywords: endocannabinoids, 2-AG, AEA, conditioned fear, innate fear, looming shadow

*Corresponding author:
Professor of Psychiatry and Behavioral Sciences
Northwestern University
Feinberg School of Medicine
320 E Superior St, 4-490
Chicago, Illinois 60611
Sachin.patel@northwestern.edu

ABSTRACT

A hallmark of many psychiatric disorders is maladaptive and heightened fear responses to non-threatening stimuli. Adaptive defensive responses to threats involve transitions between passive behaviors, such as freezing, and active escape strategies, such as darting or fleeing. The endocannabinoid (eCB) system, particularly 2-arachidonoylglycerol (2-AG), plays a crucial role in modulating fear and stress responses. However, the extent to which 2-AG influences defensive behavioral state transitions to fear responses remains unclear. To address this, we investigated the role of 2-AG in shaping defensive behaviors to learned and innate threats using pharmacological manipulations in both the serial compound stimulus (SCS) and the looming shadow paradigm. During SCS, inhibition of 2-AG synthesis enhanced freezing to early cues and promoted active responses during cues associated with heightened threat imminence. In the looming shadow paradigm, 2-AG depletion biased defensive behavior toward freezing and increased time spent in a safe zone, suggesting a shift toward passive responses. These findings demonstrate that 2-AG signaling critically regulates the balance and transitions between passive and active defensive strategies in both learned and innate fear contexts. Thus, 2-AG plays a key role in the scaling of defensive response transitions and the promotion of active defensive responses to threats.

INTRODUCTION

The selection of appropriate defensive responses to actual and perceived threats is vital to an organism's survival (Fadok et al. 2017; Borkar et al. 2020; Le et al. 2024). However, heightened fear responses in non-threatening situations are a hallmark symptom of anxiety- and stress-related disorders. According to the predatory imminence theory, defensive behaviors shift as the proximity of a threat, both spatially and temporally, increases (Fanselow and Lester 1988; Blanchard and Blanchard 1989; Fanselow 2018). Elucidating the neurobiological mechanisms underlying these adaptive behaviors could be important for uncovering potential therapeutic targets for these highly prevalent mental disorders.

The endocannabinoid (eCB) system is a neuromodulatory system that functions to suppress neurotransmission. 2-arachidonylglycerol (2-AG), the brain's most abundant eCB, is synthesized in the postsynaptic membrane in an activity-dependent manner by the enzyme diacylglycerol lipase alpha (DAGLα). It then retrogradely activates $G_{i/o}$-coupled cannabinoid receptor 1 (CB1R) on presynaptic terminals to inhibit neurotransmitter release before being degraded by monoacylglycerol lipase (MAGL)(Kano et al. 2009). A large body of work has demonstrated the central role of 2-AG in responding to stress and threat (Riebe et al. 2012; Lutz et al. 2015; Patel et al. 2022; Gunduz-Cinar et al. 2023; Ramos-Medina et al. 2024). Additionally, augmentation of anandamide (AEA), another common eCB molecule, signaling has shown anxiolytic effects in response to stress and facilitation of fear extinction (Kathuria et al. 2003; Lafenêtre et al. 2007; Gunduz-Cinar et al. 2013; Petrie et al. 2021). However, the role of the eCB system in modulating transitions between defensive states across the spectrum of threat imminence remains elusive.

To assess learned defensive response switching, we used the serial compound stimulus (SCS) paradigm that elicits freezing followed by flight (darting/jumping) responses as the auditory

stimulus progresses from tone to white noise. Specifically, SCS consisted of a 10-second long pure tone followed immediately by 10 seconds of white noise and terminates with a strong 1-second foot shock (Fadok et al. 2017; Borkar et al. 2020; Totty et al. 2021; Le et al. 2024). This protocol allowed us to evaluate behavioral state transitions as threat imminence and salience increase during the transition from pure tone to white noise (Fadok et al. 2017). To explore innate defensive response switching we used a looming shadow paradigm (Daviu et al., 2020a; Daviu et al., 2020b; Gunduz-Cinar et al., 2013; Shang et al., 2018; Yilmaz & Meister, 2013). In this paradigm, mice are repeatedly exposed to growing overhead shadows to mimic descent by an aerial predator, to examine defensive responses spanning from freezing to the shadow to fleeing to a safe space (covered tent).

By combining these behavioral paradigms with pharmacological manipulation of 2-AG levels, this study provides insight into the role of the eCB system in mediating defensive responses across a spectrum of learned and innate threats. We hypothesize that 2-AG signaling critically influences the selection and transition between passive and active coping strategies during escalating threat imminence. Understanding how the eCB system modulates these behaviors can deepen our knowledge of the neurobiological mechanisms underlying adaptive and maladaptive responses to fear and stress, ultimately guiding the development of targeted therapies for anxiety- and stress-related disorders.

MATERIALS AND METHODS

Animal Care

Male and female C57BL/6J mice aged 8-12 weeks were used for all behavioral experiments. Animals were group-housed (five animals per cage) on a 12-h light/dark cycle with food and water

provided ad libitum. Experiments were conducted during the light phase. Mice were given a one-week acclimation period to the facilities and handled by experimenters before testing. All experiments were carried out according to guidelines provided by Northwestern University Animal Care and Use Committee.

Drug Treatment

All treatments were injected intraperitoneally (I.P.) 2 hours before testing on conditioning days for the fear conditioning paradigms and 2 hours before looming shadow testing. DO34 (50 mg/kg, Glixx Laboratories) was dissolved in an 18:1:1 solution of saline (Hospira, Inc.), ethanol (Decon Laboratories, Inc.), and kolliphor EL (Sigma-Aldrich). Vehicle treatment consisted of the 18:1:1 solution only. JZL-184 (10 mg/kg, Cayman Chemical Company) or PF-3845 (1 mg/kg, Cayman Chemical Company) were dissolved in 100% dimethylsulfoxide (DMSO; 1 µl/g, Sigma-Aldrich), with 100% DMSO as the corresponding vehicle (Morgan et al. 2022; Kondev et al. 2022, 2023a).

SCS Paradigm

The serial compound stimulus (SCS) conditioned flight protocol was used as previously described (Fadok et al. 2017). Two contexts were used for the SCS paradigm. Context A was a half-circle chamber made of a multi-colored plexiglass wall and floor. The behavior room lights were dimmed. Red tape was added to lights within the chamber to alter the lighting, and a vanilla scent was used to further distinguish Context A. Context B was a rectangular chamber (38cm x 19cm x 30cm) with metal floor grids housed within soundproof boxes (Coulbourn Instruments). 70% ethanol was used to clean both contexts. Auditory stimuli were delivered at 75 dB via speakers within the boxes above the chambers and foot shocks were delivered via the metal grids using FreezeFrame software (Actimetrics). On all days, animals were given a 3-minute habituation period to the context before the onset of the first auditory stimulus. On Day 0, four SCS pairings

of 10s pure tone (7.5 kHz) pips (500 ms at 1 Hz) and 10s white noise pips (500 ms at 1 Hz) were delivered in context A with pseudorandom intertrial intervals (ITIs) of 50-90s. On Days 1 and 2, five pairings of SCS were delivered in context B with pseudorandom ITIs of 150-210s, followed immediately by a 1s shock (0.9 mA). On Day 3, sixteen pairings of SCS were delivered in context B with pseudorandom ITIs of 60-100s for fear extinction.

Escape Score Calculations

The escape score was calculated as a combination of speed differences between experimental phases and the number of jumps observed during each phase. First, the mean speed during the Pre-SCS (baseline), Pure Tone (PT), and White Noise (WN) phases was computed for each trial. To stabilize variance, all speed values were transformed using a natural logarithm, with a small constant (epsilon) added to avoid undefined values. The speed difference for each phase was then calculated by subtracting the log-transformed Pre-SCS speed from the log-transformed speeds during the PT and WN phases, reflecting changes in activity relative to baseline. The number of jumps during the PT and WN phases was directly added to their respective speed differences to account for escape-related motor events. This approach yielded escape scores that integrate both locomotor and discrete escape behaviors.

1. Speed Data Preparation:

   Pre-SCS period (Pre-SCS): The mean speed during the -10 to 0 seconds interval.

   Pure Tone (PT) period: The mean speed during the 1 to 10s interval.

   White Noise (WN) period: The mean speed during the 11 to 20s interval.

2. Log Transformation: applied to speed values to reduce skewness of data

   $$\text{LogSpeed}_{\text{Pre-SCS}} = \ln(\text{Speed}_{\text{Pre-SCS}} + \epsilon)$$

   $$\text{LogSpeed}_{\text{PT}} = \ln(\text{Speed}_{\text{PT}} + \epsilon)$$

$$LogSpeed_{WN} = \ln(Speed_{WN} + \epsilon)$$

3. Speed Difference Calculation:

$$SpeedDiff_{PT} = LogSpeed_{PT} - LogSpeed_{Pre\text{-}SCS}$$

$$SpeedDiff_{WN} = LogSpeed_{WN} - LogSpeed_{Pre\text{-}SCS}$$

4. Escape Score Calculation

$$EscapeScore_{PT} = SpeedDiff_{PT} + Jumps_{PT}$$

$$EscapeScore_{WN} = SpeedDiff_{WN} + Jumps_{WN}$$

Looming Shadow Paradigm

Behavior was tested in a clear rectangular arena (40 cm x 20 cm x 16 cm) with a red plexiglass shelter on one end of the space. A red light was placed outside of the arena on the tent side to see the mouse inside the tent. A rear-end projection screen was suspended 30 cm above the base of the arena and a projector (Elephas) sat 66 cm above the screen to provide projection of the looming shadow stimuli. A separate computer displaying the looming shadow stimuli was connected to the projector. Shadow stimuli consisted of five cycles of a black circle growing over 500 ms, with the total duration lasting 3 seconds. Stimuli were triggered manually by the experimenter when the animal entered a predefined area on the opposite end of the cage from the shelter. Animal behavior was recorded using ANY-maze.

Subjects were given 15 minutes to habituate to the arena prior to the onset of the looming shadow stimuli. The animals then underwent a 15-minute testing stage, in which they were presented with a shadow when the animal entered the pre-determined “far zone” (20 cm x 8 cm) on the opposite end of the arena. After the shadow presentation, a one-minute inter-presentation interval was implemented during which no shadow stimuli were triggered, irrespective of the animal's location. Testing was concluded after the subject executed five shadow presentations or

upon the expiration of the 15-minute testing period. 70% ethanol was used to clean the arena between animals.

Behavioral Analyses

For fear conditioning, freezing behavior was quantified using Freezeframe, and animal speed was quantified using ANY-maze with the animal's centroid tracked for analysis purposes. All behaviors were binned into 1s bins. An independent investigator blind to treatment hand-scored all jump data.

For looming shadow, an independent investigator blind to treatment hand-scored the length of time an animal spent in the tent and the latency to respond to each shadow. Defensive responses were categorized as no response, freeze, dart (fleeing to tent immediately after shadow presentation), freeze-to-dart (freezing then fleeing to the tent), or timed out (were unable to receive all five shadow responses in the 15-minute testing period due to excessive time in the tent).

Statistical Analyses

All statistical analyses were performed with Prism 10 (GraphPad, San Diego, CA, USA) and MATLAB R2024a (MathWorks, Natick, MA, USA) software. Normality was determined using the Shapiro-Wilk test. Group effects were analyzed using paired Student's t-test or analysis of variance (ANOVA), depending on the number of independent variables. When correcting for multiple comparisons, post-hoc analyses of ANOVAs included Holm-Šídák's or Dunnett's test. Otherwise, Fisher's Least Significant Difference (LSD) was used. Chi-squared analyses were conducted in MATLAB. Details for each analysis can be found in Tables 1-7. $P<0.05$ was considered significant throughout.

RESULTS

Pharmacological inhibition of 2-AG synthesis affects both passive and active defensive responses in the SCS paradigm

SCS paradigm consisted of a pre-exposure (habituation) day followed by two days of conditioning and one day of extinction (Fig. 1). SCS presentation on the pre-exposure day did not induce freezing or flight responses (Supplemental Fig. 1, Table 4). To test the effect of 2-AG signaling on active flight responses (fleeing and jumping) versus passive (freezing) responses to the SCS, we pharmacologically inhibited 2-AG synthesis with DO34 (50 mg/kg) 2 hours before both days of conditioning (Fig. 2A, Table 1). Across both days, DO34-treated mice exhibited higher freezing during PT and lower freezing during WN compared to vehicle-treated mice [stimulus × treatment interaction: Day 1, $F (39, 312) = 2.192$, $P=0.0001$ (Fig. 2C); Day 2, $F (39, 312) = 3.602$, $P<0.0001$ (Fig. 2J), Table 1]. Conversely, speed was reduced during PT and increased during WN for DO34-treated mice [stimulus × treatment interaction: Day 1, $F (1, 16) = 11.26$, $P=0.0040$ (Fig. 2E); $F (1, 16) = 9.490$, $P=0.0072$ (Fig. 2L), Table 1].

We also analyzed jumping behavior as a specific subset of flight responses between vehicle and DO34-treated mice. Jumping behavior was significantly influenced by treatment [main effect of treatment: Day 1, $F(1,16) = 7.902$, $P = 0.0125$; Day 2, $F (1, 16) = 4.167$, $P=0.0581$ (Fig. 2F, 2M), Table 1] and interacted with trial and stimulus context [Day 1, treatment × trials × stimulus interaction: $F(4,64) = 3.588$, $P = 0.0106$] (Fig. 2E, Table 1). DO34-treated mice exhibited more jumps during WN compared to vehicle-treated controls (Fig. 2G, Table 1). To further quantify flight responses, escape scores were calculated using speed and jump data ((Fadok et al. 2017; Hersman et al. 2020; Borkar et al. 2020; Le et al. 2024); see *Materials and Methods*). DO34-treated mice had significantly higher escape scores than vehicle-treated mice during WN on both days

[Day 1, P = 0.0164; Day 2, P = 0.0462], while vehicle-treated mice displayed higher escape scores during PT on Day 2 (P = 0.0429) (Fig. 2G, 2N, Table 1).

Next, we wanted to determine whether enhancing eCB levels could reduce the magnitude of defensive responses to SCS. Thus, mice were treated with JZL-184 (inhibitor of the 2-AG degradation enzyme, MAGL), PF-3845 (inhibitor of the AEA degradation enzyme, FAAH), or vehicle. Freezing differences between PT and WN were observed across all groups, but no significant treatment effects were detected (Supplemental Fig. 3, Table 5). On Day 1, JZL-184-treated mice displayed increased speed during WN [main effect of treatment, $P < 0.05$], but this effect disappeared by Day 2, with stimulus type driving speed differences across groups (Supplemental Fig. 3, Table 5).

To assess the lasting effects of DAGL inhibition during conditioning on extinction of SCS-driven behavior, mice underwent a single extinction session in the same conditioning context drug-free, receiving 16 SCS presentations without shock. Extinction learning showed no significant interaction between stimulus type and treatment for freezing, speed, or jumps across all trials (Fig. 3A-E, Table 2). To evaluate potential differences in extinction rates, trials were divided into early (trials 1–4) and late (trials 13–16) stages. During early extinction, previously DO34-treated mice exhibited slower speeds [Sidak's post-hoc, P = 0.0433 (Fig. 3G, Table 2)] and fewer jumps during WN [Sidak's post-hoc, P = 0.0162 (Fig. 3H, Table 2)]. DO34-treated mice also had marginally higher escape scores during WN, reflecting divergence from baseline active responses rather than overall activity levels. Despite persistent differences in freezing and speed between PT and WN, no treatment effects were detected.

The impact of enhanced eCB signaling during acquisition on extinction was also assessed. Similar to 2-AG attenuation, a main effect of stimulus type on freezing and speed was observed

during extinction. However, treatment effects were absent during early extinction and when averaging all trials. Notably, a main effect of treatment on speed emerged during late extinction trials (F (2, 17) = 4.765, P=0.0228) (Supplemental Fig. 5G, Table 6).

Pharmacological inhibition of 2-AG synthesis biases towards passive defensive behaviors during innate threat

To test whether 2-AG signaling affects defensive response strategies in response to innate threat, mice were treated with vehicle or DO34 and underwent a looming shadow protocol that elicits both active and passive defensive responses (Daviu et al., 2020a; Daviu et al., 2020b). In an open arena with a tent “safe zone”, mice were exposed to up to five presentations of an overhead, growing circular shadow simulating an aerial predator (Fig. 4A-B, see *Materials and Methods*). DO34-treated mice spent significantly more time in the tent compared to vehicle-treated controls during the testing phase (Fig. 4C), though there were no differences in response latency to the shadow presentations (Fig. 4D). Notably, vehicle-treated mice exhibited more instances of no response to the shadows, whereas DO34-treated mice timed out significantly more often (Fig. 4E, Table 3, Supplemental Fig. 6). These findings suggest that 2-AG deficiency is associated with enhanced passive defensive responses during repeated innate threat presentation.

Next, we examined whether augmenting eCB signaling affected defensive responses to the looming shadow. Similar to the DO34-treated mice, there were no differences in the latency to respond to shadows between vehicle- JZL-184-, and PF-3845-treated groups (Supplemental Fig. 7A, Table 4). Additionally, there were no differences in the amount of time spent in the tent between the three treatment groups (Supplemental Fig. 7B, Table 4). However, comparisons between vehicle versus PF-3845 revealed a significantly lower proportion of 'No Response' in the

PF-3845 group ($\chi^2 = 12.89$, $p = 0.0003$, Table 7) and a significantly higher proportion of 'Dart' responses ($\chi^2 = 6.38$, $p = 0.0115$, Table 7). No other categories differed significantly between these groups. In contrast, vehicle versus JZL-184 comparisons revealed no significant differences were found across individual response categories (all $p > 0.05$, Table 7). These findings suggest that increased AEA levels may promote active state defense responses to innate threats.

DISCUSSION

The appropriate selection of behavioral responses in dangerous situations is vital for survival and is influenced by the perceived proximity and intensity of threats. Various studies have investigated the biological underpinnings surrounding the four most common responses to a perceived threat: fight, flight, freeze, and fawn (Zingela et al. 2022). The mechanisms underlying shifts between different response states, however, remain incompletely understood. The eCB has emerged as a key modulator of threat appraisal and defensive behavior selection (Maldonado et al. 2020). The present study extends these findings by delineating the role of the eCB system in shaping passive-to-active behavioral transitions in response to both learned and innate aversive stimuli.

To discern the role of 2-AG in regulating passive (freezing) and active (jumping, fleeing) defensive behaviors, we first employed the SCS paradigm. On day 1 of conditioning, pharmacological reduction of 2-AG via DO34 treatment led to an increased number of jumps during the WN component compared to vehicle-treated mice. Interestingly, on Day 2 we observed that DO34-treatment led to heightened passive responses to the pure tone *and* increased active responses to white noise. It is important to contextualize these findings within the overall freezing patterns across conditioning. On Day 1, average pre-SCS freezing values were elevated

(~40%) for both treatment groups and freezing during the pure tone and white noise periods did not significantly exceed this baseline. However, freezing levels during trial 1 were comparable to those on Day 0 (Supplemental Fig. 2), suggesting that initial elevated freezing reflects a generalized fear response to the first tone-shock pairing rather than a drug-induced locomotor impairment. This interpretation is further supported by the similar pre-SCS freezing levels between DO34 and vehicle groups, and prior evidence that DO34 does not impair general locomotion (Ramos-Medina et al., 2024). While cue-specific freezing was limited on Day 1, clear cue discrimination emerged by Day 2 and during extinction, indicating intact associative learning. These findings suggest that 2-AG depletion may facilitate fear memory consolidation, thereby amplifying defensive responses during subsequent SCS exposure.

Extinction of conditioned fear responses is also essential for adaptive behavior, enabling reductions in defensive responses when threat is no longer present. Impaired extinction is a hallmark of stress-related disorders, including PTSD (Norrholm et al. 2011). Prior work has demonstrated that diminished eCB signaling can impair fear extinction (Marsicano et al. 2002; Hill et al. 2018; Cavener et al. 2018; Ramos-Medina et al. 2024), whereas enhancement of eCB signaling is linked to facilitation of fear extinction (Chhatwal et al. 2005, 2009; Bitencourt et al. 2008; Gunduz-Cinar et al. 2013, 2023). Interestingly, we found that during early extinction (trials 1-4), previously DO34-treated mice showed significantly attenuated active fear responses (Fig. 3G, H). However, their calculated escape scores were higher than controls, which appears to be a limitation of the metric itself: if a mouse exhibited complete freezing during the pre-SCS period, the resulting score was artificially inflated due to a near-zero denominator (Supplemental Fig. 6). These results suggest that 2-AG may promote fear extinction by preferentially dampening active defensive responses. The neural underpinnings of these effects may involve

circuit-specific modulation, as two different cell types in the central amygdala – somatostatin and corticotropin-releasing factor neurons– initiate passive freezing behavior and mediate conditioned flight responses, respectively (Fadok et al. 2017). Thus, we conclude that 2-AG depletion during conditioning promotes the subsequent extinction of active defensive responses to SCS presentation, however the effects of 2-AG depletion during extinction training itself were not evaluated.

In contrast, global enhancement of 2-AG levels via MAGL inhibition (JZL-184) did not have significant effects on fear acquisition or extinction. These findings partially align with a previous work from our lab in which JZL-184 pre-treatment had no impact on cued-conditioning fear learning (Hartley et al. 2016). However, while the former study saw impairment in fear extinction, we did not replicate that effect with our SCS protocol. This discrepancy may stem from the timings of the JZL-184 administration. In the present study, our findings inform us of the effects of varying eCB tone during fear acquisition on extinction learning, as drug treatments only occurred on conditioning days. Meanwhile, our prior work examined the effects of enhanced 2-AG levels during extinction learning and, thus, drug treatments were delivered one hour prior to extinction sessions (Hartley et al. 2016).

Similarly, augmenting AEA levels via FAAH inhibition (PF-3845) did not alter fear learning or extinction. One possible explanation is that naturally occurring AEA signaling may be maximally engaged during conditioning, and further elevations in AEA content cannot affect defensive response generation. This is supported by studies that show increased AEA levels in the medial prefrontal cortex, amygdala, hippocampus, and periaqueductal gray following footshock stress (Hohmann et al. 2005; Morena et al. 2014). However, some studies have demonstrated contrasting results with reductions in AEA content globally (Bluett et al. 2014) and in the amygdala

(Vecchiarelli et al. 2022) after footshock stress. Thus, these findings highlight the complexity of AEA signaling dynamics in response to stress, suggesting that its effects on defensive behavior may be region-specific, temporally regulated, and highly sensitive to experimental context.

Taken together, these data indicate that 2-AG deficiency is associated with progressively enhanced magnitude of passive and active responses to SCS across conditioning days, thereby amplifying both defensive response strategies to SCS presentation. However, analysis of escape scores suggests DO34 treatment may bias behavior toward active coping as threat imminence increases, driving a transition from passive to active responses. These data point to an important role for endogenous 2-AG in constraining both active and passive defensive responses during escalating threat imminence.

Adaptive responses to predator-like threats generally fall into two primary categories: (1) avoiding detection through behaviors like freezing and (2) avoiding capture through actions such as fleeing or fighting (Yilmaz and Meister 2013; Shang et al. 2018; Salay et al. 2018). In this study, we used a looming shadow paradigm to elicit these behaviors and examine how eCBs regulate the repertoire and shift of behaviors to a perceived imminent threat. Consistent with previous reports, we observed that the predominant defense responses to looming shadow stimuli were flight/escape behaviors, including darting and transitions from freezing to darting (Fig. 4D) (Yilmaz and Meister 2013; Daviu et al. 2020b). Our results showed that pharmacological inhibition of 2-AG synthesis led to animals spending significantly more time in the tent, with no changes in latency to respond to the shadows. Additionally, DO34-treated mice timed-out significantly more than vehicle-treated mice, suggesting exaggerated passive defensive responses at the expense of exploratory behavior during the inter-trial interval when no threat was present. While we did not analyze behavior as a function of time across the looming stimulus itself, doing

so in future work could reveal whether 2-AG dynamically modulates the shift from passive to active responses as the perceived threat escalates. This would offer further insight into how 2-AG regulates adaptive defense strategies in a threat-imminence-dependent manner. Taken together, endogenous 2-AG may be important for terminating passive defensive responses in the absence of threat and promoting optimal exploratory behavior in the absence of threat.

Importantly, these effects appear to be specific to reductions in 2-AG, as enhancing 2-AG levels via the MAGL inhibitor JZL-184 had no significant effect on shadow-evoked behaviors, latency to respond, or time spent in the tent. In contrast, augmenting AEA levels by blocking its degradation promoted more passive behavioral responses, although latency and time in tent remained unaffected. Our data suggest that 2-AG signaling may be necessary for adaptive behavioral transitions in the absence of threat, while AEA may serve to modulate coping style toward more passive responding without altering threat detection or appraisal. These results align with previous work in which mice showed reduced flight behaviors to an approaching robo-beetle after enhancement of AEA levels (Heinz et al. 2017). Similarly, we observed fewer darts and decreased reactivity to shadow stimuli, indicative of a shift toward more passive coping strategies. However, while they also reported that enhanced 2-AG signaling increased flight responses(Heinz et al. 2017), which we did not observe. Notably, their study lacked a designated ‘safe zone’ for the mice to retreat to, which may have influenced the expression of escape responses. This difference in task design may explain the divergent behavioral outcomes and suggests that environmental context can shape how 2-AG modulates threat responses.

One alternative interpretation is that eCBs induce alterations in visual perception that affect defense responses. While it is well-established that eCBs are known to play important roles in visual system development (Bouchard et al. 2016), less is known about the acute effects

of 2-AG depletion on visual processing. However, a recent study examined how different cannabinoids affect mouse visual acuity. They report that pharmacological augmentation and attenuation of 2-AG decreased and increased visual acuity, respectively, while augmenting AEA did not affect acuity (Cécyre et al. 2020). This aligns with our finding that DO34 does not impair threat detection, as treated mice displayed normal latencies to respond to the looming stimulus, suggesting intact visual processing. Future studies directly assessing visual function during acute 2-AG depletion will be necessary to fully rule out this possibility.

Beyond behavioral outcomes, future studies should also dissect the neural circuitry by which 2-AG signaling shapes defensive state transitions. Selective targeting of eCB machinery using promising tools such as the $DAGL\alpha^{f/f}$ (Winters et al.) or $CB1^{f/f}$ (Marcus et al. 2020; Kondev et al. 2023b) mouse lines may provide insight into how local eCB tone modulates defensive behavior. Notably, recent work has demonstrated that the visual cortex can instruct the suppression of innate defensive responses through a top-down pathway to the ventrolateral geniculate nucleus (vLGN), with this learning-induced plasticity requiring eCB-mediated long-term suppression of inhibitory synapses onto vLGN neurons (Mederos et al. 2025). Such findings reinforce the idea that eCB signaling is critical for adaptive behavioral plasticity in response to complex and evolving threat environments.

In summary, our study demonstrates that 2-AG signaling plays a critical role in regulating behavioral state transitions across both learned and innate fear responses. During learned fear paradigms, such as the SCS, endogenous 2-AG appears to constrain the magnitude of both passive and active defense strategies, potentially promoting adaptive responses to escalating threat imminence. Similarly, in innate fear paradigms like the looming shadow test, endogenous 2-AG appears to promote exploratory behavior between threat presentations and thus may serve

an adaptive role to maximize exploratory/foraging in the absence of threat. These findings highlight the context-dependent role of the endocannabinoid system in facilitating appropriate behavioral selection during perceived threats. By revealing how 2-AG shapes the repertoire and transition of defensive states, this study underscores the broader impact of the eCB system on adaptive responses to fear and stress. Elucidating these mechanisms may ultimately inform novel therapeutic strategies for fear dysregulation in stress-related psychiatric disorders.

REFERENCES

Bitencourt RM, Pamplona FA, Takahashi RN (2008) Facilitation of contextual fear memory extinction and anti-anxiogenic effects of AM404 and cannabidiol in conditioned rats. European Neuropsychopharmacology 18:849–859. https://doi.org/10.1016/j.euroneuro.2008.07.001

Blanchard RJ, Blanchard DC (1989) Antipredator defensive behaviors in a visible burrow system. Journal of Comparative Psychology 103:70–82. https://doi.org/10.1037/0735-7036.103.1.70

Bluett RJ, Gamble-George JC, Hermanson DJ, et al (2014) Central anandamide deficiency predicts stress-induced anxiety: behavioral reversal through endocannabinoid augmentation. Transl Psychiatry 4:e408–e408. https://doi.org/10.1038/tp.2014.53

Borkar CD, Dorofeikova M, Le QSE, et al (2020) Sex differences in behavioral responses during a conditioned flight paradigm. Behavioural Brain Research 389:. https://doi.org/10.1016/j.bbr.2020.112623

Bouchard J-F, Casanova C, Cécyre B, Redmond WJ (2016) Expression and Function of the Endocannabinoid System in the Retina and the Visual Brain. Neural Plast 2016:9247057. https://doi.org/10.1155/2016/9247057

Cavener VS, Gaulden A, Pennipede D, et al (2018) Inhibition of Diacylglycerol Lipase Impairs Fear Extinction in Mice. Frontiers in Neuroscience 12:. https://doi.org/10.3389/FNINS.2018.00479/FULL

Cécyre B, Bachand I, Papineau F, et al (2020) Cannabinoids affect the mouse visual acuity via the cannabinoid receptor type 2. Sci Rep 10:15819. https://doi.org/10.1038/s41598-020-72553-y

Chhatwal JP, Davis M, Maguschak KA, Ressler KJ (2005) Enhancing Cannabinoid Neurotransmission Augments the Extinction of Conditioned Fear. Neuropsychopharmacol 30:516–524. https://doi.org/10.1038/sj.npp.1300655

Chhatwal JP, Gutman AR, Maguschak KA, et al (2009) Functional Interactions between Endocannabinoid and CCK Neurotransmitter Systems May Be Critical for Extinction Learning. Neuropsychopharmacol 34:509–521. https://doi.org/10.1038/npp.2008.97

Daviu N, Füzesi T, G Rosenegger D, et al (2020a) Visual-looming Shadow Task with in-vivo Calcium Activity Monitoring to Assess Defensive Behaviors in Mice. Bio Protoc 10:e3826. https://doi.org/10.21769/BioProtoc.3826

Daviu N, Füzesi T, Rosenegger DG, et al (2020b) Paraventricular nucleus CRH neurons encode stress controllability and regulate defensive behavior selection. Nat Neurosci 23:398–410. https://doi.org/10.1038/s41593-020-0591-0

Fadok JP, Krabbe S, Markovic M, et al (2017) A competitive inhibitory circuit for selection of active and passive fear responses. Nature 542:96–99. https://doi.org/10.1038/nature21047

Fanselow MS (2018) The Role of Learning in Threat Imminence and Defensive Behaviors. Curr Opin Behav Sci 24:44–49. https://doi.org/10.1016/j.cobeha.2018.03.003

Fanselow MS, Lester LS (1988) A functional behavioristic approach to aversively motivated behavior: Predatory imminence as a determinant of the topography of defensive behavior. In: Evolution and learning. Lawrence Erlbaum Associates, Inc, Hillsdale, NJ, US, pp 185–212

Gunduz-Cinar O, Castillo LI, Xia M, et al (2023) A cortico-amygdala neural substrate for endocannabinoid modulation of fear extinction. Neuron 111:3053-3067.e10. https://doi.org/10.1016/j.neuron.2023.06.023

Gunduz-Cinar O, MacPherson KP, Cinar R, et al (2013) Convergent translational evidence of a role for anandamide in amygdala-mediated fear extinction, threat processing and stress-reactivity. Mol Psychiatry 18:813–823. https://doi.org/10.1038/mp.2012.72

Hartley ND, Gunduz-Cinar O, Halladay L, et al (2016) 2-arachidonoylglycerol signaling impairs short-term fear extinction. Transl Psychiatry 6:e749–e749. https://doi.org/10.1038/tp.2016.26

Heinz DE, Genewsky A, Wotjak CT (2017) Enhanced anandamide signaling reduces flight behavior elicited by an approaching robo-beetle. Neuropharmacology 126:233–241. https://doi.org/10.1016/j.neuropharm.2017.09.010

Hersman S, Allen D, Hashimoto M, et al (2020) Stimulus salience determines defensive behaviors elicited by aversively conditioned serial compound auditory stimuli. eLife 9:1–26. https://doi.org/10.7554/eLife.53803

Hill MN, Campolongo P, Yehuda R, Patel S (2018) Integrating Endocannabinoid Signaling and Cannabinoids into the Biology and Treatment of Posttraumatic Stress Disorder. Neuropsychopharmacol 43:80–102. https://doi.org/10.1038/npp.2017.162

Hohmann AG, Suplita RL, Bolton NM, et al (2005) An endocannabinoid mechanism for stress-induced analgesia. Nature 435:1108–1112. https://doi.org/10.1038/nature03658

Kano M, Ohno-Shosaku T, Hashimotodani Y, et al (2009) Endocannabinoid-mediated control of synaptic transmission. Physiological Reviews 89:309–380. https://doi.org/10.1152/PHYSREV.00019.2008/ASSET/IMAGES/LARGE/Z9J0010924970013.JPEG

Kathuria S, Gaetani S, Fegley D, et al (2003) Modulation of anxiety through blockade of anandamide hydrolysis. Nat Med 9:76–81. https://doi.org/10.1038/nm803

Kondev V, Morgan A, Najeed M, et al (2022) The Endocannabinoid 2-Arachidonoylglycerol Bidirectionally Modulates Acute and Protracted Effects of Predator Odor Exposure. Biological Psychiatry 92:739–749. https://doi.org/10.1016/j.biopsych.2022.05.012

Kondev V, Najeed M, Loomba N, et al (2023a) Synaptic and cellular endocannabinoid signaling mechanisms regulate stress-induced plasticity of nucleus accumbens somatostatin neurons. Proceedings of the National Academy of Sciences of the United States of America 120:e2300585120. https://doi.org/10.1073/pnas.2300585120

Kondev V, Najeed M, Yasmin F, et al (2023b) Endocannabinoid release at ventral hippocampal-amygdala synapses regulates stress-induced behavioral adaptation. Cell Reports 42:113027. https://doi.org/10.1016/j.celrep.2023.113027

Lafenêtre P, Chaouloff F, Marsicano G (2007) The endocannabinoid system in the processing of anxiety and fear and how CB1 receptors may modulate fear extinction. Pharmacol Res 56:367–381. https://doi.org/10.1016/j.phrs.2007.09.006

Le Q-SE, Hereford D, Borkar CD, et al (2024) Contributions of associative and non-associative learning to the dynamics of defensive ethograms. eLife 12:. https://doi.org/10.7554/eLife.90414.2

Lutz B, Marsicano G, Maldonado R, Hillard CJ (2015) The endocannabinoid system in guarding against fear, anxiety and stress. Nature reviews Neuroscience 16:705. https://doi.org/10.1038/NRN4036

Maldonado R, Cabañero D, Martín-García E (2020) The endocannabinoid system in modulating fear, anxiety, and stress. Dialogues Clin Neurosci 22:229–239. https://doi.org/10.31887/DCNS.2020.22.3/rmaldonado

Marcus DJ, Bedse G, Gaulden AD, et al (2020) Endocannabinoid Signaling Collapse Mediates Stress-Induced Amygdalo-Cortical Strengthening. Neuron 105:1062-1076.e6. https://doi.org/10.1016/j.neuron.2019.12.024

Marsicano G, Wotjak CT, Azad SC, et al (2002) The endogenous cannabinoid system controls extinction of aversive memories. Nature 418:530–534. https://doi.org/10.1038/nature00839

Mederos S, Blakely P, Vissers N, et al (2025) Overwriting an instinct: Visual cortex instructs learning to suppress fear responses. Science 387:682–688. https://doi.org/10.1126/science.adr2247

Morena M, Roozendaal B, Trezza V, et al (2014) Endogenous cannabinoid release within prefrontal-limbic pathways affects memory consolidation of emotional training. Proceedings of the National Academy of Sciences 111:18333–18338. https://doi.org/10.1073/pnas.1420285111

Morgan A, Adank D, Johnson K, et al (2022) 2-Arachidonoylglycerol-mediated endocannabinoid signaling modulates mechanical hypersensitivity associated with alcohol withdrawal in mice. Alcohol Clin Exp Res 46:2010–2024. https://doi.org/10.1111/acer.14949

Norrholm SD, Jovanovic T, Olin IW, et al (2011) Fear Extinction in Traumatized Civilians with Posttraumatic Stress Disorder: Relation to Symptom Severity. Biological Psychiatry 69:556–563. https://doi.org/10.1016/j.biopsych.2010.09.013

Patel S, Johnson K, Adank D, Rosas-Vidal LE (2022) Longitudinal monitoring of prefrontal cortical ensemble dynamics reveals new insights into stress habituation. Neurobiol Stress 20:100481. https://doi.org/10.1016/j.ynstr.2022.100481

Petrie GN, Nastase AS, Aukema RJ, Hill MN (2021) Endocannabinoids, cannabinoids and the regulation of anxiety. Neuropharmacology 195:108626. https://doi.org/10.1016/j.neuropharm.2021.108626

Ramos-Medina L, Rosas-Vidal LE, Patel S (2024) Pharmacological diacylglycerol lipase inhibition impairs contextual fear extinction in mice. Psychopharmacology 241:569–584. https://doi.org/10.1007/s00213-023-06523-3

Riebe CJ, Pamplona FA, Kamprath K, Wotjak CT (2012) Fear relief-toward a new conceptual frame work and what endocannabinoids gotta do with it. Neuroscience 204:159–185. https://doi.org/10.1016/j.neuroscience.2011.11.057

Salay LD, Ishiko N, Huberman AD (2018) A midline thalamic circuit determines reactions to visual threat. Nature 557:183–189. https://doi.org/10.1038/s41586-018-0078-2

Shang C, Chen Z, Liu A, et al (2018) Divergent midbrain circuits orchestrate escape and freezing responses to looming stimuli in mice. Nat Commun 9:1232. https://doi.org/10.1038/s41467-018-03580-7

Totty MS, Warren N, Huddleston I, et al (2021) Behavioral and brain mechanisms mediating conditioned flight behavior in rats. Scientific Reports 11:8215. https://doi.org/10.1038/s41598-021-87559-3

Vecchiarelli HA, Morena M, Lee TTY, et al (2022) Sex and stressor modality influence acute stress-induced dynamic changes in corticolimbic endocannabinoid levels in adult Sprague Dawley rats. Neurobiology of Stress 20:100470. https://doi.org/10.1016/j.ynstr.2022.100470

Winters ND, Bedse G, Astafyev AA, et al Targeting diacylglycerol lipase reduces alcohol consumption in preclinical models. J Clin Invest 131:e146861. https://doi.org/10.1172/JCI146861

Yilmaz M, Meister M (2013) Rapid innate defensive responses of mice to looming visual stimuli. Current Biology 23:2011–2015. https://doi.org/10.1016/J.CUB.2013.08.015/ATTACHMENT/69F4EDAC-71B7-410C-9348-E4B193222CD5/MMC3.MP4

Zingela Z, Stroud L, Cronje J, et al (2022) The psychological and subjective experience of catatonia: a qualitative study. BMC Psychology 10:173. https://doi.org/10.1186/s40359-022-00885-7

ETHICAL APPROVAL

All experiments were carried out according to guidelines provided by Northwestern University Animal Care and Use Committee.

DATA AVAILABILITY STATEMENT

N/A

AUTHOR CONTRIBUTIONS

N.L., A.C., and S.P. designed research; N.L., A.C., I.K., M.K., and S.C. performed research; N.L., A.C., and S.C. analyzed the data; N.L., A.C., and S.P. wrote the paper.

COMPETING INTERESTS

All authors declare no conflicts of interest.

FUNDING

These studies were supported by the National Institutes of Health grant MH107435 (S.P.) and the National Science Foundation Graduate Research Fellowship Program (N.L.).

MAIN FIGURES

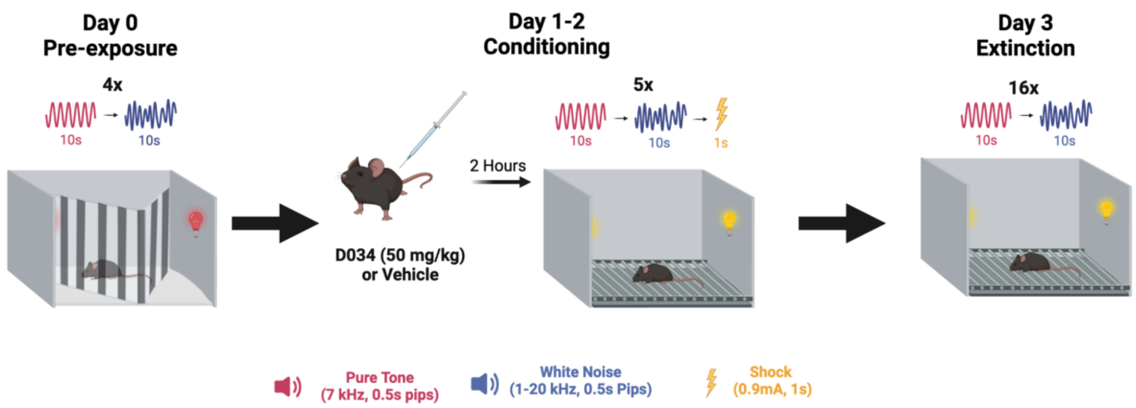

Figure 1. Schematic of Serial Compound Stimulus Paradigm (SCS). The experimental timeline includes three phases: Day 0 (Pre-exposure), Days 1–2 (Conditioning), and Day 3 (Extinction). On Day 0, mice are exposed to alternating presentations of a pure tone (7 kHz, 0.5-second pips) and white noise (1–20 kHz, 0.5-second pips) for 10 seconds each, repeated four times with pseudorandom intertrial intervals. On Days 1–2, mice receive an intraperitoneal injection of DO34 (50 mg/kg) or vehicle 2 hours prior to conditioning. During conditioning, the auditory stimuli are presented in the same sequence, with each trial ending in a 1-second foot shock (0.9 mA). The sequence is repeated five times. On Day 3, mice undergo extinction trials consisting of 16 repetitions of the auditory stimuli without foot shocks.

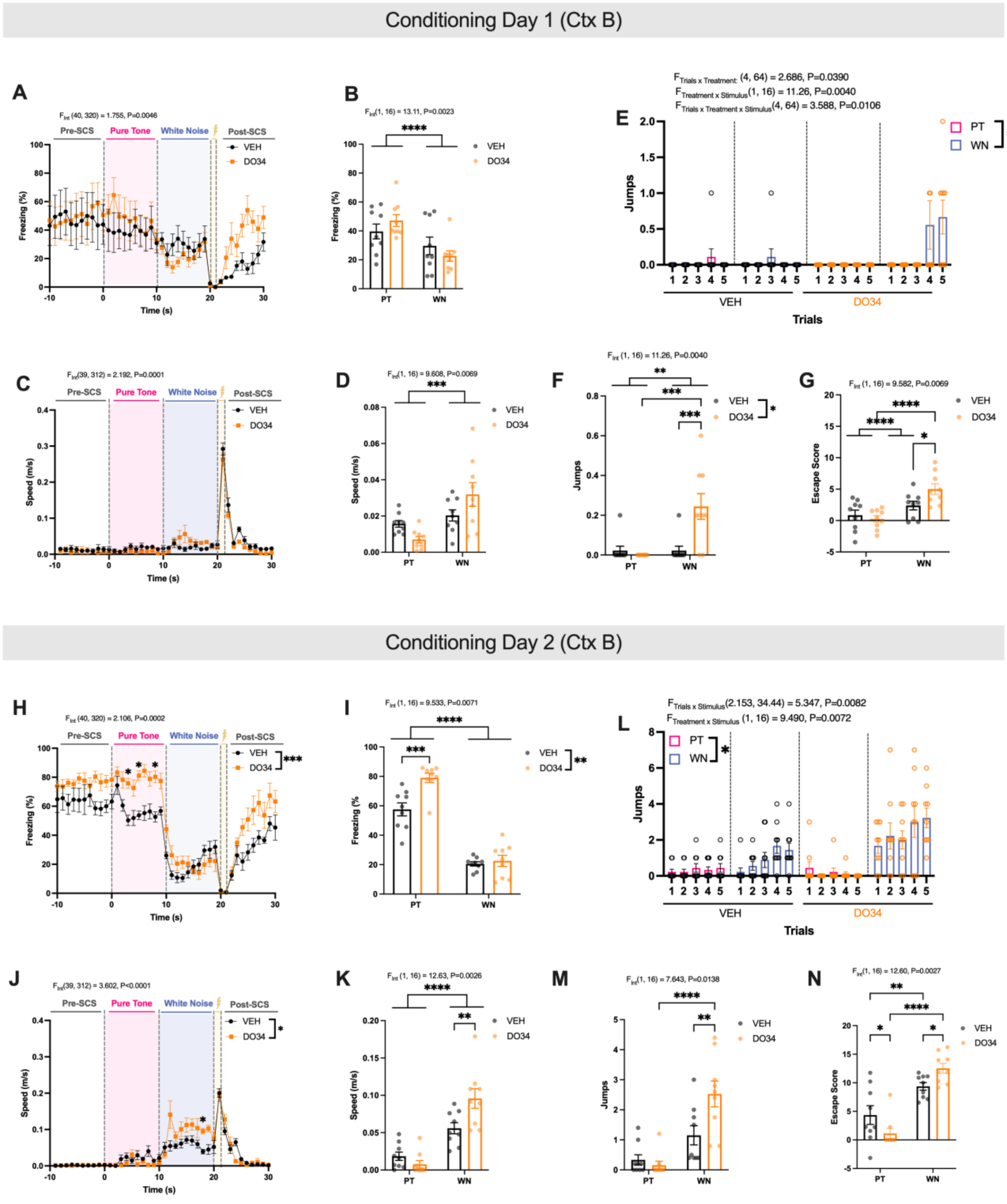

Figure 2. Attenuating 2-AG on conditioning days heightens freezing and flight behavior. (A) Schematic of serial compound stimulus (SCS) paradigm (B-H) Data are from conditioning day 1. (I-O) Data are from conditioning day 2. (B,I) Average freezing trace of vehicle- (VEH, n= 4 males, 5 females) and DO34-(n=4 males, 5 females) treated mice across SCS presentations. (C, J) Average freezing during PT and WN by treatment group. (D, K) Average speed trace of VEH and DO34 treated mice across SCS presentations. (E, L) Average speed during PT and WN by

treatment group. (F, M) Number of jumps during PT and WN per trial. (G, N) Average number of jumps across all trials during PT and WN by treatment group. (H, O) Average escape scores across all trials during PT and WN by treatment group. All values are presented as mean ± SEM. *$p<0.05$, **$p<0.01$, ***$p<0.001$, ****$p<0.0001$.

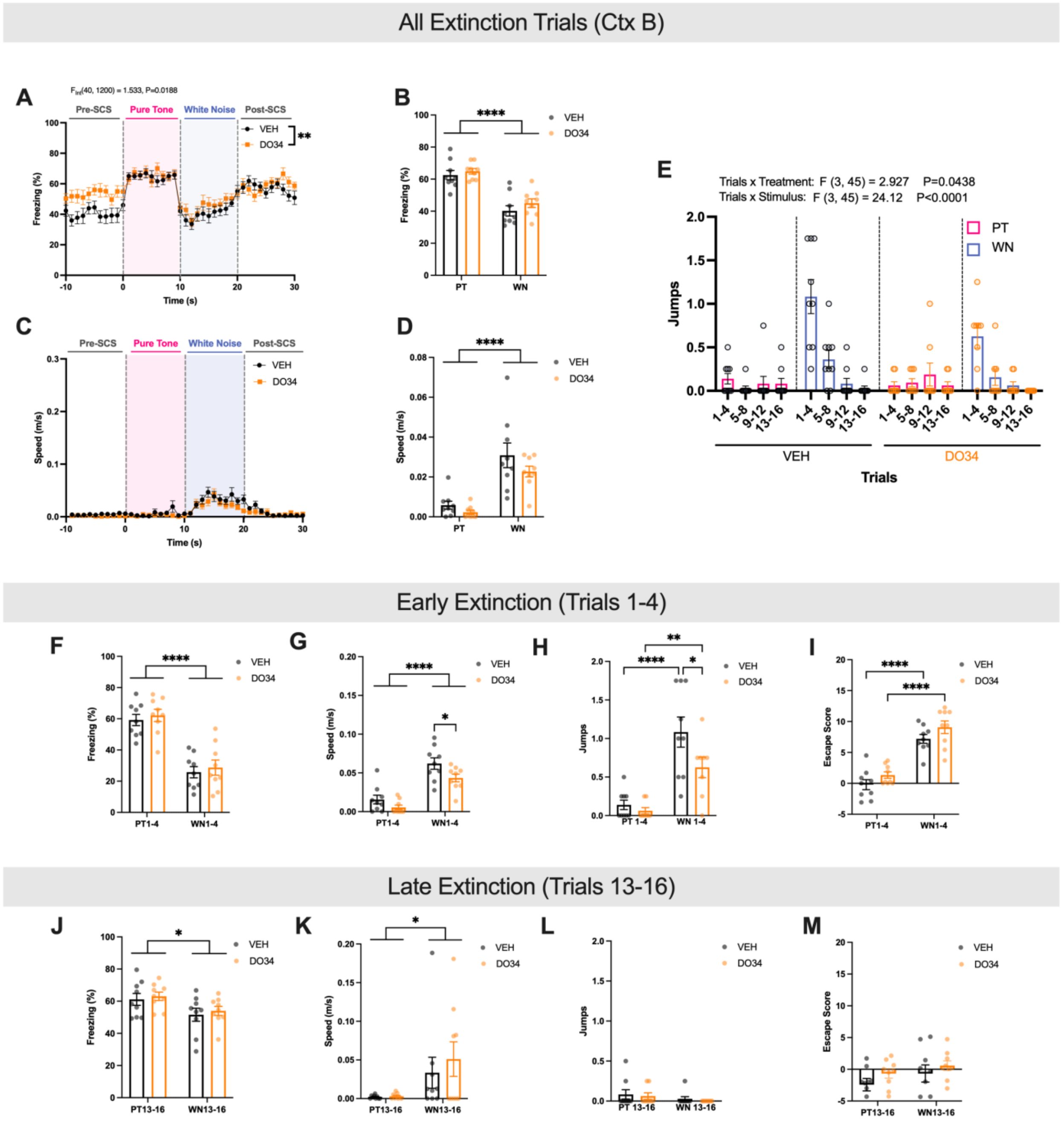

Fig 3. Attenuating 2-AG during fear acquisition has lasting effects on escape behavior during early extinction. (A-E) Data are averages from all trials on extinction day. (A) Average freezing trace of vehicle- (VEH, n=4 males, 5 females) and DO34-(n=4 males, 5 females) treated mice across all 16 SCS presentations. (B) Average freezing during PT and WN by treatment group. (C) Average speed trace of VEH and DO34 treated mice across SCS presentations. (D) Average speed during PT and WN by treatment group. (E) Average number of jumps during PT and WN per every four trials. (F-I) Data are averages from early extinction trials (Trials 1-4). (J-M) Data are averages from late extinction trials (Trials 13-16). (F, J) Average freezing during PT and WN by treatment group. (G, K) Average speed during PT and WN. (H, L) Average number of jumps during PT and WN by treatment group. (I, M) Average escape scores during PT and

WN by treatment group. All values are presented as mean ± SEM. $*p<0.05$, $**p<0.01$, $***p<0.001$, $****p<0.0001$.

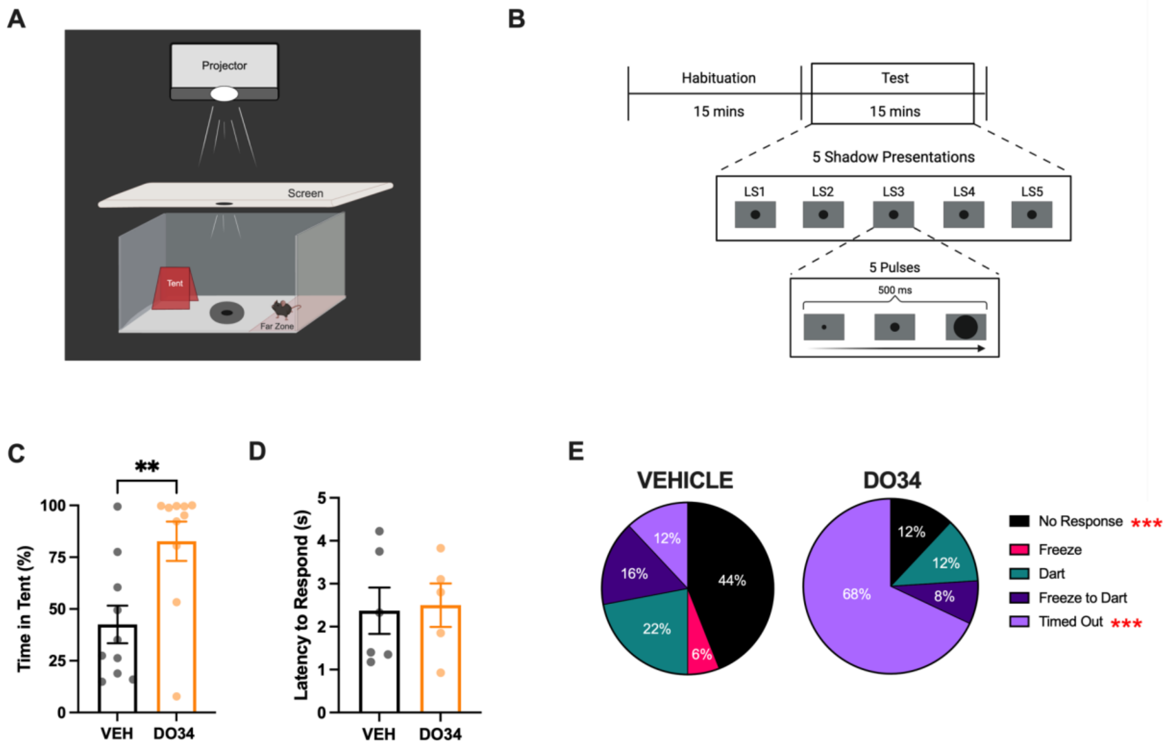

Fig 4. Attenuating 2-AG biases passive state responses to innate fear. (A) Looming shadow arena (B) Behavioral paradigm (C) Percent time spent in tent during test stage. (D) Average latency to respond to shadow presentation. (E) Average percentage of times a behavior was displayed in response to shadow by all mice in each treatment group (VEH: n=5 males, 5 females; DO34: n=5 males, 5 females). Differences between treatment groups were determined by chi-squared test. All error bars represented as ± SEM. *p<0.05, **p<0.01, ***p<0.001, ****p<0.0001.

SUPPLEMENTAL FIGURES

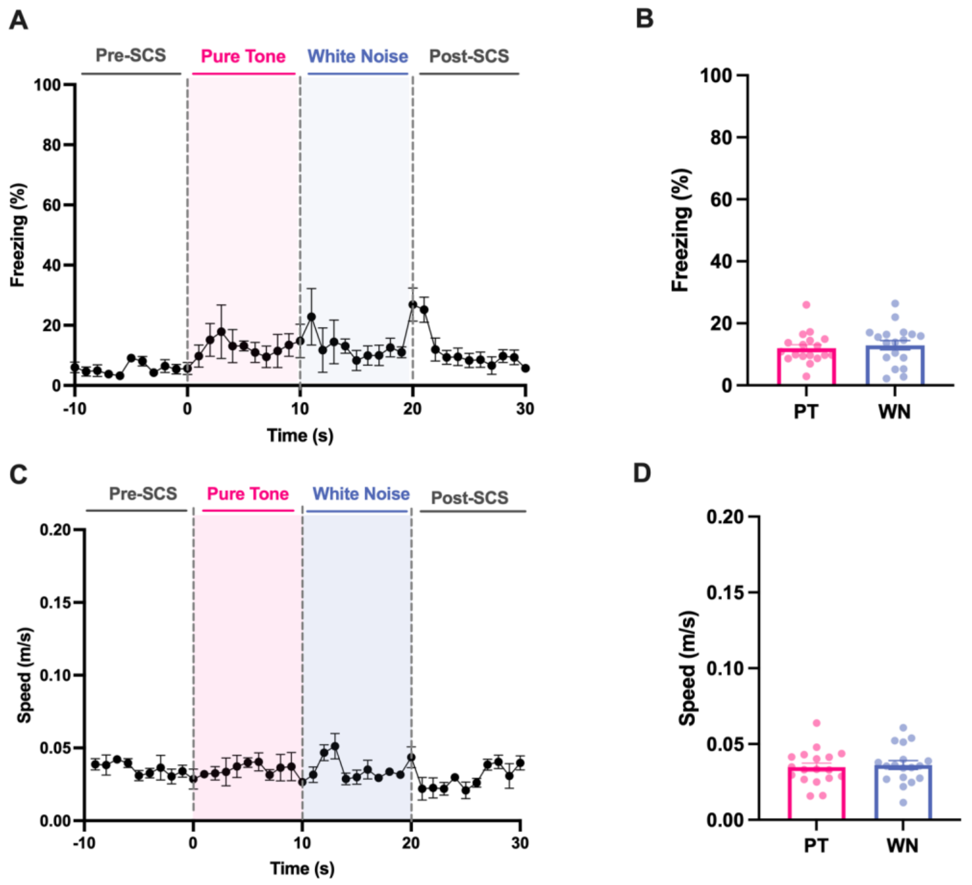

Supplemental Figure 1. Baseline behavioral responses to pure tone versus white noise. (A) Average freezing across 4 SCS trials (n=8 males, 10 females). (B) Average freezing per mouse to pure tone and white noise. (C) Average speed across 4 SCS trials. (D) Average speed per mouse to pure tone and white noise.

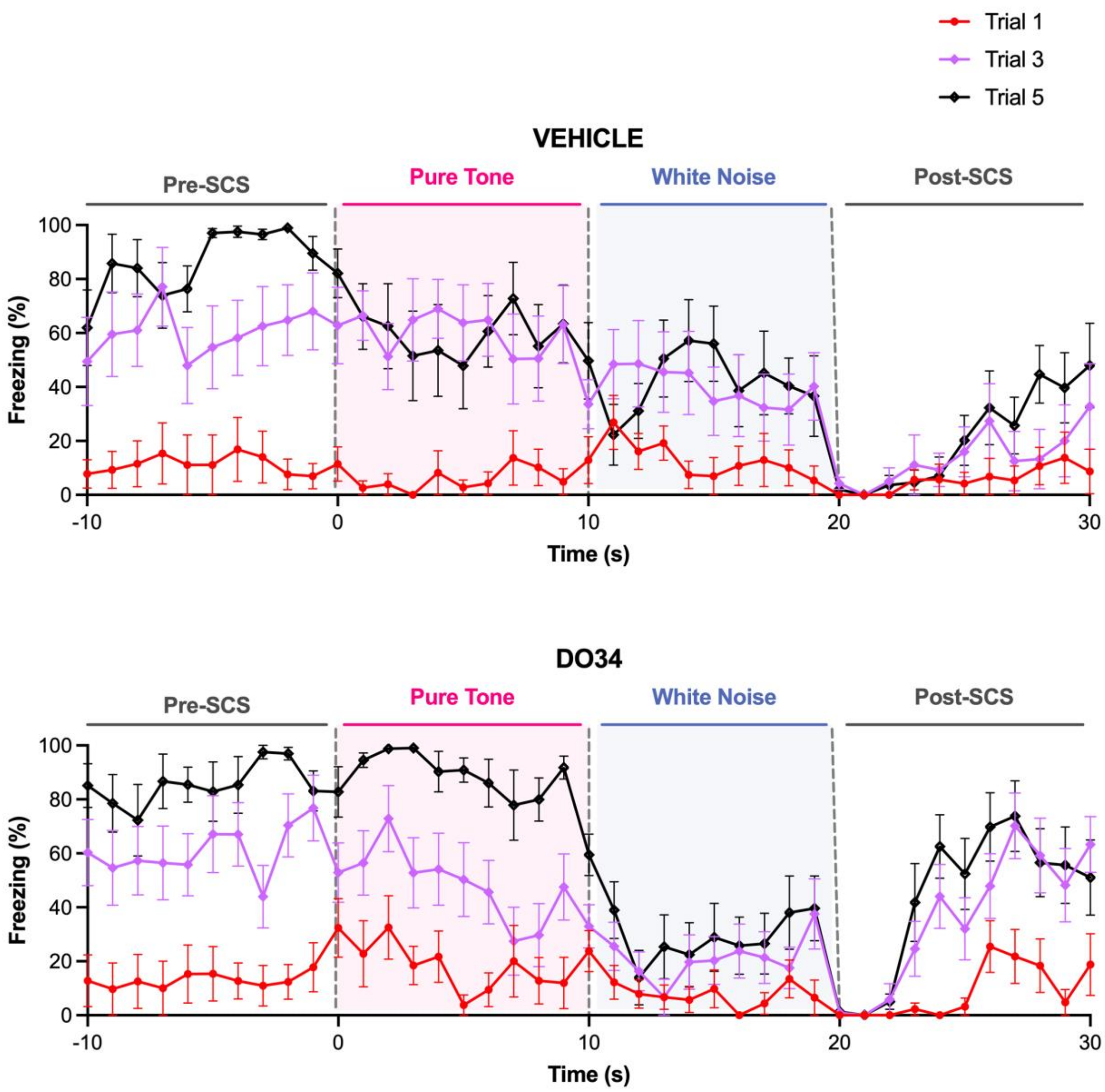

Supplemental Figure 2. Freezing across trials during SCS conditioning day 1. Average freezing levels of vehicle-treated (top, n= 4 males, 5 females) and DO34-treated (bottom, n=4 males, 5 females) across trials 1 (red), 3 (purple), and 5 (black) of SCS Day 1. All values are presented as mean ± SEM.

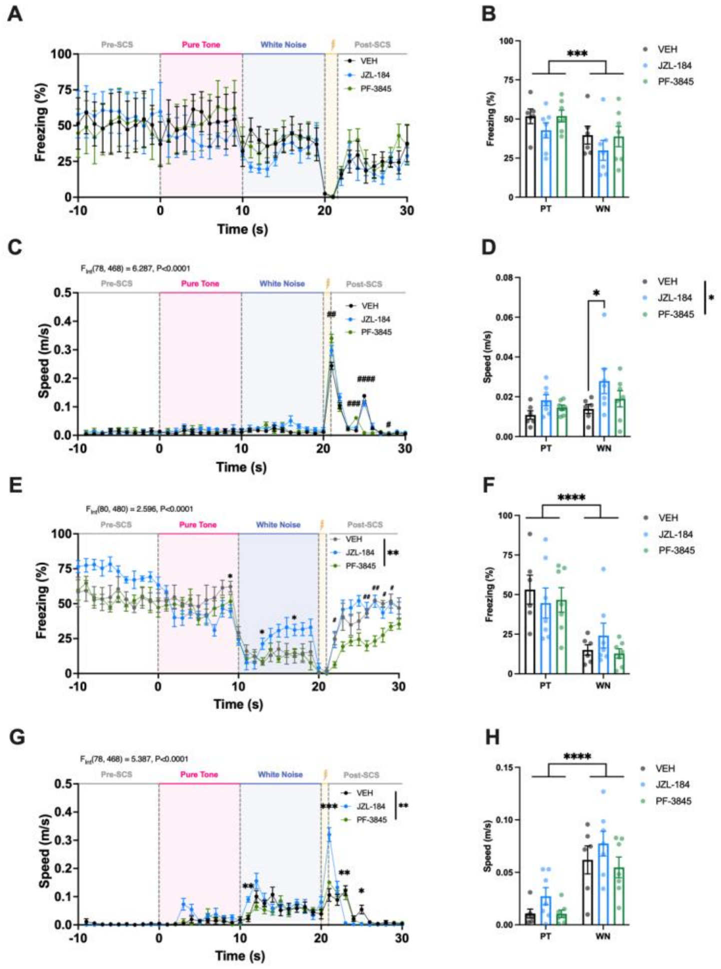

Supplemental Figure 3. Augmenting 2-AG and AEA does not alter defensive responses to PT and WN during fear acquisition. (A-D) Data are from conditioning day 1. (E-H) Data are from conditioning day 2. (A, E) Average freezing trace of vehicle (VEH, n=3 males, 3 females), JZL-184 (n=4 males, 3 females), and PF-3845 (n=3 males, 4 females) groups across all SCS trials. (B, F) Average freezing during PT and WN by treatment group. (D, H) Average speed trace of VEH and DO34 treated mice across SCS presentations. (C, G) Average speed during PT and WN by treatment group. All values are presented as mean ± SEM. *p<0.05, **p<0.01, ***p<0.001, ****p<0.0001.

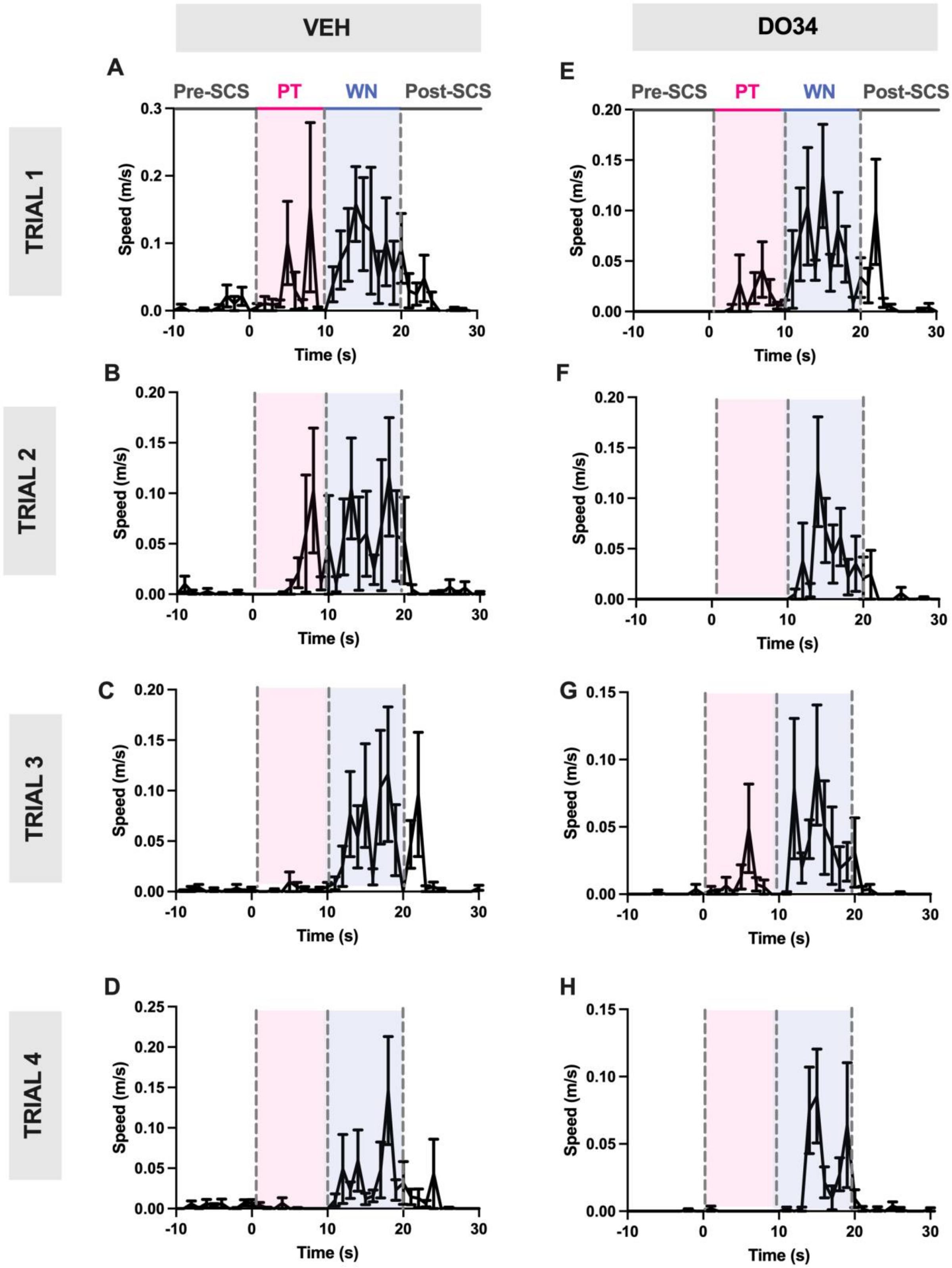

Supplemental Figure 4. Early extinction speed traces. (A-D) Speed trace of first 4 extinction trials in vehicle-treated mice (n=4 males, 5 females). (E-H) Speed trace of first 4 extinction trials in DO34-treated mice (n=4 males, 5 females).

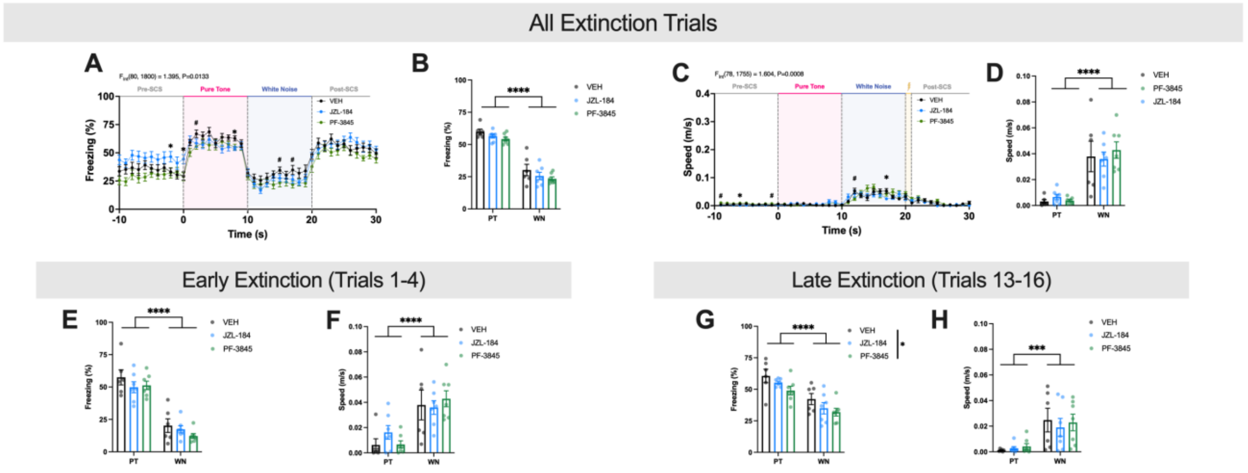

Supplemental Figure 5. Augmenting 2-AG and AEA does not alter responses to SCS during extinction. (A-D) Data are averages from all trials on extinction day. (A) Average freezing trace of vehicle, JZL-184, and PF-3845 groups across all 16 SCS presentations. (B) Average freezing during PT and WN by treatment group. (C) Average speed trace of VEH (n=3 males, 3 females), JZL-184 (n=4 males, 3 females), and PF-3845 (n=3 males, 4 females) groups across all 16 SCS presentations. (D) Average speed during PT and WN by treatment group. (E, F) Data are averages from early extinction trials (Trials 1-4). (G, H) Data are averages from late extinction trials (Trials 13-16). (E, G) Average freezing during PT and WN by treatment group. (F, H) Average speed during PT and WN by treatment group. All error bars represented as ± SEM. *p<0.05, **p<0.01, ***p<0.001, ****p<0.0001.

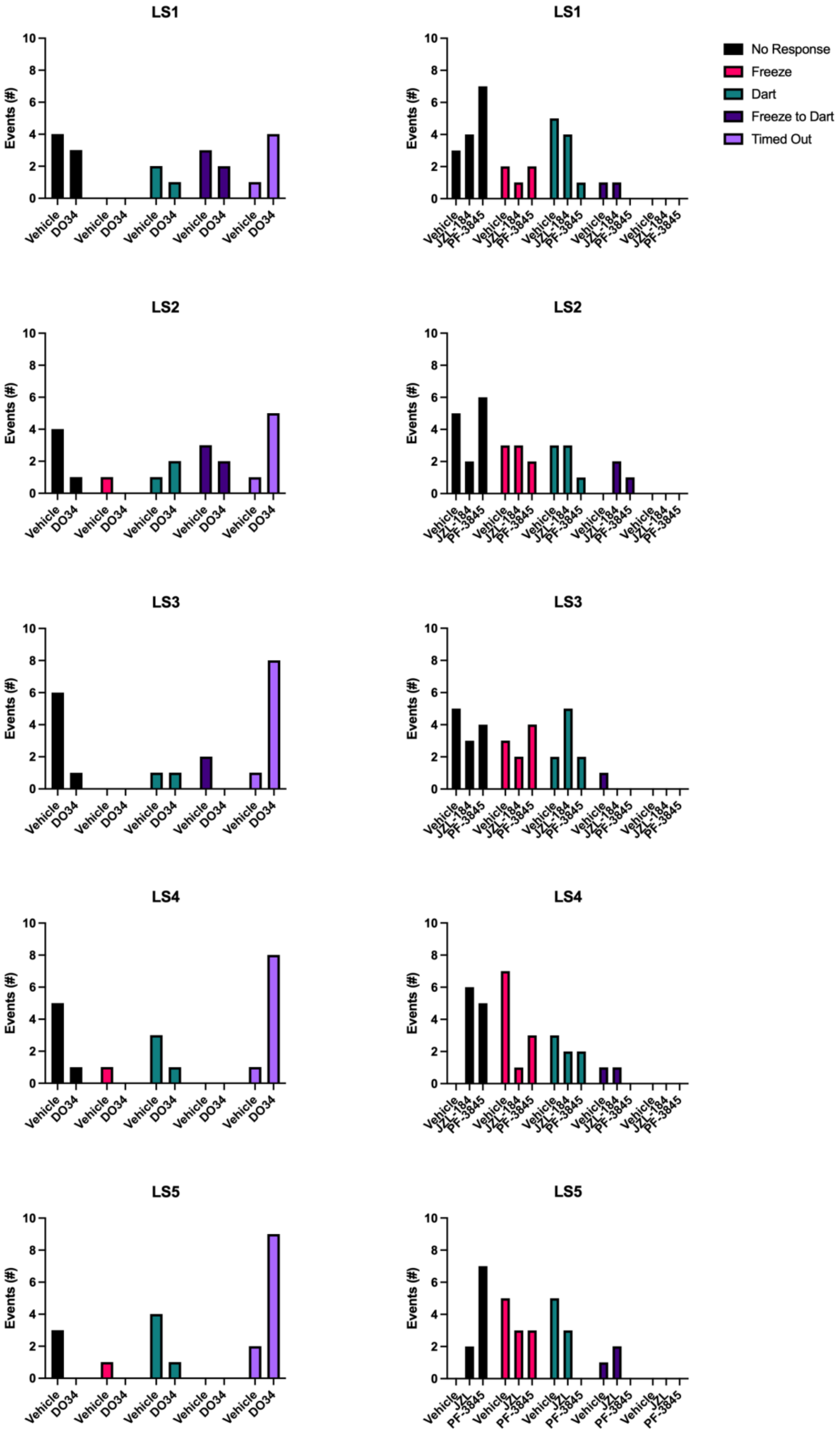

LS1
LS2
LS3
LS4
LS5
Events (#)
Vehicle
DO34
JZL-184
JZL
PF-3845
No Response
Freeze
Dart
Freeze to Dart
Timed Out

Supplemental Figure 6. Behavioral responses to looming shadows. Description of behavioral response events for each shadow presentation (LS1-LS5) across vehicle and DO34 treatments (left; VEH: n=5 males, 5 females; DO34: n= 5 males, 5 females) and vehicle, JZL-184, and PF-3845 treatments (right; VEH: n=6 males, 5 females; JZL-184: n=5 males, 5 females; PF-3845: n=5 males, 5 females).

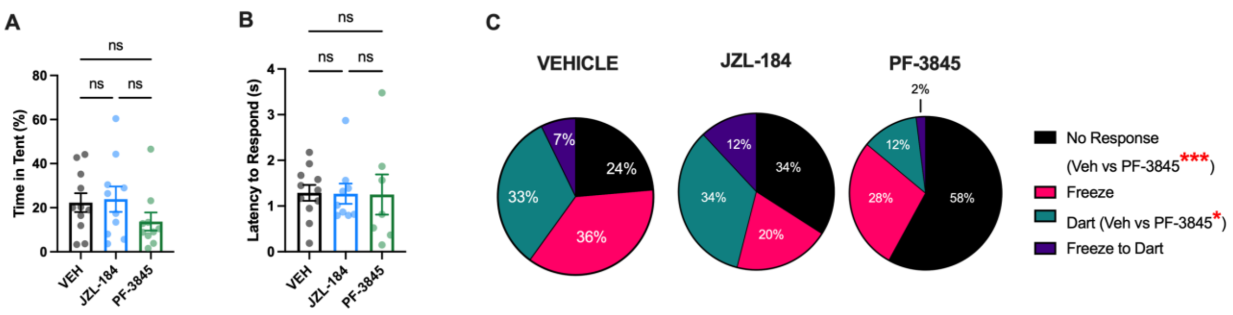

Supplemental Figure 7. Responses to looming shadow following 2-AG or AEA augmentation. (A) Percent time spent in tent during the test stage. (B) Average latency to respond to shadow presentation (C) Average percentage of times a behavior was displayed in response to shadow by all mice in each treatment group (VEH: n=5 males, 5 females; JZL-284: n= 5 males, 5 females; PF-3845: n= 5 males, 5 females). Differences between vehicle and drug treatment groups were determined by chi-squared test. All error bars represented as ± SEM.

TABLES

Table 1

Figure	Statistical Test	Post-hoc Analysis	Main Effects	Interactions
**2A**	2-way RM ANOVA	Sidak's multiple comparisons	Stimulus: F (40, 320) = 8.719, P<0.0001 Treatment: F (1, 8) = 0.2010, P=0.6658	F (40, 320) = 1.755, P=0.0046
**2B**	2-way RM ANOVA	Sidak's multiple comparisons	Stimulus: F (1, 16) = 73.28, P<0.0001 Treatment: F (1, 16) = 0.001781, P=0.9669	F(1, 16) = 13.11, P=0.0023
**2C**	2-way RM ANOVA	Sidak's multiple comparisons	Stimulus: F (3.845, 30.76) = 84.45, P<0.0001 Treatment: F (1, 8) = 0.6337, P=0.4490	F (39, 312) = 2.192, P=0.0001
**2D**	2-way RM ANOVA	Sidak's multiple comparisons	Stimulus: F (1, 16) = 20.43, P=0.0003 Treatment: F (1, 16) = 0.1134, P=0.7407	F (1, 16) = 9.608, P=0.0069
**2E**	3-way ANOVA	N/A	Trials: F (4, 64) = 2.861, P=0.0303 Treatment: F (1, 16) = 7.902, P=0.0125 Stimulus: F (1, 16) = 11.26, P=0.0040	Trials x Treatment: F (4, 64) = 2.686, P=0.0390 Trials x Stimulus: F (4, 64) = 2.118, P=0.0888 Treatment x Stimulus: F (1, 16) = 11.26, P=0.0040 Trials x Treatment x Stimulus: F (4, 64) = 3.588, P=0.0106
**2F**	2-way RM ANOVA	Fisher's LSD	Stimulus: F (1, 16) = 11.26, P=0.0040 Treatment: F (1, 16) = 7.902, P=0.0125	F (1, 16) = 11.26, P=0.0040
**2G**	2-way RM ANOVA	Fisher's LSD	Stimulus: F (1, 16) = 36.81, P<0.0001 Treatment: F (1, 16) = 1.277, P=0.2751	F (1, 16) = 9.582, P=0.0069
**2H**	2-way RM ANOVA	Sidak's multiple comparisons	Stimulus: F (4.994, 39.95) = 42.59, P<0.0001 Treatment: F (1, 8) = 32.47, P=0.0005	F (40, 320) = 2.106, P=0.0002
**2I**	2-way RM ANOVA	Sidak's multiple comparisons	Stimulus: F (1, 16) = 220.4, P<0.0001	F (1, 16) = 9.533, P=0.0071

			Treatment: F (1, 16) = 10.61, P=0.0049	
**2J**	2-way RM ANOVA	Sidak's multiple comparisons	Stimulus: F (4.752, 38.01) = 37.38, P<0.0001<br>Treatment: F (1, 8) = 6.234, P=0.0371	F (39, 312) = 3.602, P<0.0001
**2K**	2-way RM ANOVA	Sidak's multiple comparisons	Stimulus: F (1, 16) = 77.87, P<0.0001<br>Treatment: F (1, 16) = 2.314, P=0.1477	F (1, 16) = 12.63, P=0.0026
**2L**	3-way ANOVA	N/A	Trials: F (2.698, 43.17) = 3.407, P=0.0298<br>Treatment: F (1, 16) = 5.465, P=0.0327<br>Stimulus: F (1.000, 16.00) = 29.29, P<0.0001	Trials x Treatment: F (4, 64) = 0.2169, P=0.9281<br>Trials x Stimulus: F (2.153, 34.44) = 5.347, P=0.0082<br>Treatment x Stimulus: F (1, 16) = 9.490, P=0.0072<br>Trials x Treatment x Stimulus: F (4, 64) = 0.5252, P=0.7176
**2M**	2-way RM ANOVA	Fisher's LSD	Stimulus: F (1, 16) = 32.12, P<0.0001<br>Treatment: F (1, 16) = 4.167, P=0.0581	F (1, 16) = 7.643, P=0.0138
**2N**	2-way RM ANOVA	Fisher's LSD	Stimulus: F (1, 16) = 83.52, P<0.0001<br>Treatment: F (1, 16) = 0.0004679, P=0.9830	F (1, 16) = 12.60, P=0.0027

Table 2

Figure	Statistical Test	Post-hoc Analysis	Main Effects	Interactions
**3A**	2-way RM ANOVA	Sidak's multiple comparisons	Stimulus: F(6.527, 195.8) = 13.92, P<0.0001 Treatment: F (1, 30) = 11.39, P=0.0021	F (40, 1200) = 1.533, P=0.0188
**3B**	2-way RM ANOVA	Sidak's multiple comparisons	Stimulus: F (1, 16) = 73.94, P<0.0001 Treatment: F (1, 16) = 1.552, P=0.2308	F (1, 16) = 0.2341, P=0.6351
**3C**	2-way RM ANOVA	Sidak's multiple comparisons	Stimulus: F(6.527, 195.8) = 13.92, P<0.0001 Treatment: F (1, 30) = 2.669, P=0.1128	F (39, 1170) = 1.209, P=0.1790
**3D**	2-way RM ANOVA	Sidak's multiple comparisons	Stimulus: F (1, 16) = 71.37, P<0.0001 Treatment: F (1, 16) = 1.894, P=0.1877	F (1, 16) = 0.7362, P=0.4035
**3E**	3-way ANOVA	Tukey's multiple comparisons	Trials: F (3, 45) = 24.74, P<0.0001 Treatment: F (1, 15) = 1.607, P=0.2243 Stimulus: F (1, 15) = 19.94, P=0.0005	Trials x Treatment: F (3, 45) = 2.927, P=0.0438 Trials x Stimulus: F (3, 45) = 24.12, P<0.0001 Treatment x Stimulus: F (1, 15) = 4.459, P=0.0519 Trials x Treatment x Stimulus: F (3, 45) = 1.104, P=0.3572
**3F**	2-way RM ANOVA	Sidak's multiple comparisons	Stimulus: F (1, 16) = 82.40, P<0.0001 Treatment: F (1, 16) = 0.4634, P=0.5058	F (1, 16) = 5.440e-006, P=0.9982
**3G**	2-way RM ANOVA	Sidak's multiple comparisons	Stimulus: F (1, 16) = 142.2, P<0.0001 Treatment: F (1, 16) = 4.364, P=0.0530	F (1, 16) = 1.457, P=0.2449
**3H**	2-way RM ANOVA	Fisher's LSD	Stimulus: F (1, 15) = 36.61, P<0.0001 Treatment: F (1, 15) = 4.250, P=0.0570	F (1, 15) = 2.352, P=0.1460
**3I**	2-way RM ANOVA	Fisher's LSD	Stimulus: F (1, 16) = 105.6, P<0.0001 Treatment: F (1, 16) = 4.220, P=0.0567	F (1, 16) = 0.05035, P=0.8253

**3J**	2-way RM ANOVA	Sidak's multiple comparisons	Stimulus: F (1, 16) = 6.889, P=0.0184 Treatment: F (1, 16) = 0.4900, P=0.4940	F (1, 16) = 0.003945, P=0.9507
**3K**	2-way RM ANOVA	Sidak's multiple comparisons	Stimulus: F (1, 16) = 6.922, P=0.0182 Treatment: F (1, 16) = 0.3850, P=0.5437	F (1, 16) = 0.3069, P=0.5872
**3L**	2-way RM ANOVA	Fisher's LSD	Stimulus: F (1, 15) = 4.636, P=0.0480 Treatment: F(1, 15) = 0.2471, P=0.6264	F (1, 15) = 0.01604, P=0.9009
**3M**	2-way RM ANOVA	Fisher's LSD	Stimulus: F (1, 16) = 4.320, P=0.0541 Treatment: F (1, 16) = 1.579, P=0.2269	F (1, 16) = 0.1170, P=0.7368

Table 3

Vehicle vs DO34			
**Behavior Response**	**P_0**	**Chi-squared**	**p-value**
**No Response**	0.28	12.6984	**0.0004**
**Freeze**	0.03	3.0928	0.0786
**Dart**	0.17	1.7718	0.1832
**Freeze to Dart**	0.12	1.5152	0.2184
**Timed Out**	0.4	32.6667	**1.09E-08**

Table 4

Figure	Statistical Test	p-value
**S1B**	Paired t-test	0.445
**S1D**	Paired t-test	0.6636
**S8A**	Kruskal-Wallis test	0.2566
**S8B**	Kruskal-Wallis test	0.6803

Table 5

Figure	Statistical Test	Post-hoc Analysis	Main Effects	Interactions
**S4A**	2-way RM ANOVA	Dunnet's multiple comparisons	Stimulus: F (2.664, 31.96) = 9.644, P=0.0002 Treatment: F (2, 12) = 0.05652, P=0.9453	F (80, 480) = 0.7208, P=0.9641
**S4B**	2-way RM ANOVA	Sidak's multiple comparisons	Stimulus: F (1, 16) = 73.28, P<0.0001 Treatment: F (1, 16) = 0.001781, P=0.9669	F (2, 17) = 0.01132, P=0.9887
**S4C**	2-way RM ANOVA	Dunnet's multiple comparisons	Stimulus: F (5.909, 70.90) = 183.0, P<0.0001 Treatment: F (2, 12) = 0.5522, P=0.5896	F (78, 468) = 6.287, P<0.0001
**S4D**	2-way RM ANOVA	Sidak's multiple comparisons	Stimulus: F (1, 17) = 4.375, P=0.0518 Treatment: F (2, 17) = 3.632, P=0.0486	F (2, 17) = 0.5506, P=0.5866
**S4E**	2-way RM ANOVA	Dunnet's multiple comparisons	Stimulus: F (7.207, 86.49) = 42.52, P<0.0001 Treatment: F (2, 12) = 7.732, P=0.0070	F (80, 480) = 2.596, P<0.0001
**S4F**	2-way RM ANOVA	Sidak's multiple comparisons	Stimulus: F (1, 17) = 76.51, P<0.0001 Treatment: F (2, 17) = 0.1577, P=0.8553	F (2, 17) = 2.263, P=0.1344
**S4G**	2-way RM ANOVA	Dunnet's multiple comparisons	Stimulus: F (6.817, 81.80) = 40.75, P<0.0001 Treatment: F (2, 12) = 10.95, P=0.0020	F (78, 468) = 5.387, P<0.0001
**S4H**	2-way RM ANOVA	Sidak's multiple comparisons	Stimulus: F (1, 17) = 84.88, P<0.0001 Treatment: F (2, 17) = 1.781, P=0.1985	F (2, 17) = 0.1775, P=0.8389

Table 6

Figure	Statistical Test	Post-hoc Analysis	Main Effects	Interactions
**S6A**	2-way RM ANOVA	Dunnet's multiple comparisons	Stimulus: F (8.243, 370.9) = 37.38, P<0.0001 Treatment: F (2, 45) = 15.93, P<0.0001	F (80, 1800) = 1.395, P=0.0133
**S6B**	2-way RM ANOVA	Sidak's multiple comparisons	Stimulus: F (1, 17) = 227.5, P<0.0001 Treatment: F (2, 17) = 3.250, P=0.0638	F (2, 17) = 0.03739, P=0.9634
**S6C**	2-way RM ANOVA	Dunnet's multiple comparisons	Stimulus: F (11.15, 501.7) = 26.92, P<0.0001 Treatment: F (2, 45) = 1.782, P=0.1799	F (78, 1755) = 1.604, P=0.0008
**S6D**	2-way RM ANOVA	Sidak's multiple comparisons	Stimulus: F (1, 17) = 62.86, P<0.0001 Treatment: F (2, 17) = 0.1120, P=0.8947	F (2, 17) = 0.4493, P=0.6455
**S6E**	2-way RM ANOVA	Dunnet's multiple comparisons	Stimulus: F (1, 17) = 214.3, P<0.0001 Treatment: F (2, 17) = 1.204, P=0.3243	F (2, 17) = 0.6706, P=0.5244
**S6F**	2-way RM ANOVA	Sidak's multiple comparisons	Stimulus: F (1, 17) = 51.20, P<0.0001 Treatment: F (2, 17) = 0.1348, P=0.8748	F (2, 17) = 1.533, P=0.2443
**S6G**	2-way RM ANOVA	Dunnet's multiple comparisons	Stimulus: F (1, 17) = 31.70, P<0.0001 Treatment: F (2, 17) = 4.765, P=0.0228	F (2, 17) = 0.08543, P=0.9185
**S6H**	2-way RM ANOVA	Sidak's multiple comparisons	Stimulus: F (1, 17) = 22.58, P=0.0002 Treatment: F (2, 17) = 0.1223, P=0.8857	F (2, 17) = 0.2607, P=0.7736

Table 7

	Vehicle vs JZL-184			Vehicle vs PF-3845		
**Behavior Response**	**P_0**	**Chi-squared**	**p-value**	**P_0**	**Chi-squared**	**p-value**
**No Response**	0.29	1.3784	0.2404	0.40	12.8864	**0.0003**
**Freeze**	0.29	3.4364	0.0638	0.32	0.8367	0.3603
**Dart**	0.33	0.0191	0.8901	0.23	6.3813	**0.0115**
**Freeze to Dart**	0.10	0.6792	0.4099	0.05	1.6055	0.2051
**Timed Out**	N/A	N/A	N/A	N/A	N/A	N/A

Adis Bohn Stafleu van Loghum palgrave macmillan Springer

Change of authorship request form (pre-acceptance)

Please read the important information on page 4 before you begin

This form should be used by authors to request any change in authorship including changes in corresponding authors. Please fully complete all sections. Use black ink and block capitals and provide each author's full name with the given name first followed by the family name.

Please note: In author collaborations where there is formal agreement for representing the collaboration, it is sufficient for the representative or legal guarantor (usually the corresponding author) to complete and sign the Authorship Change Form on behalf of all authors.

Section 1: Please provide the current title of manuscript
(For journals: Please provide the manuscript ID, title and/or DOI if available.)
(For books: Please provide the title, ISBN and/or DOI if available.)

**Manuscript ID no. in case of unpublished manuscript:**
**DOI in case of published manuscript:**
**ISBN (for books):**

Title: Endocannabinoid modulation of defensive state transitions to innate and learned threat

Section 2: Please provide the previous authorship, in the order shown on the manuscript before the changes were introduced. Please indicate the corresponding author by adding (CA) behind the name.

	First name(s)	Family name	ORCID or SCOPUS id, if available
Co-1st author	Niharika	Loomba	
Co-1st author	Anyu	Cao	
2rd author	Senna	Charles	
3rd author	Michelle	Kwon	
4th author	Sachin	Patel (CA)	
5th author			
6th author			

Please use an additional sheet if there are more than 7 authors.

SPRINGER NATURE Springer Nature is one of the world's leading global research, educational and professional publishers, created in May 2015 through the combination of Nature Publishing Group, Palgrave Macmillan, Macmillan Education and Springer Science+Business Media.

Change of authorship request form (pre-acceptance)

Section 3: Please provide a justification for change. Please use this section to explain your reasons for changing the authorship of your manuscript, e.g. what necessitated the change in authorship? Please refer to the (journal) policy pages for more information about authorship. Please explain why omitted authors were not originally included and/or why authors were removed on the submitted manuscript.

Isaac Kandil assisted in the running of additional experiments requested during revisions. He was not invovled in the original experiementation.

Section 4: Proposed new authorship. Please provide your new authorship list in the order you would like it to appear on the manuscript. Please indicate the corresponding author by adding (CA) behind the name. <u>If the corresponding author has changed, please indicate the reason under section 3.</u>

	First name(s)	Family name (this name will appear in full on the final publication and will be searchable in various abstract and indexing databases)
Co-1st author	Niharika	Loomba
Co-1st author	Anyu	Cao
2nd author	Senna	Charles
3rd author	Isaac	Kandil
4th author	Michelle	Kwon
5th author	Sachin	Patel (CA)
6th author		

Please use an additional sheet if there are more than 7 authors.

SPRINGER NATURE Springer Nature is one of the world's leading global research, educational and professional publishers, created in May 2015 through the combination of Nature Publishing Group, Palgrave Macmillan, Macmillan Education and Springer Science+Business Media.

Change of authorship request form (pre-acceptance)

Section 5: Author contribution, Acknowledgement and Disclosures. Please use this section to provide a new disclosure statement and, if appropriate, acknowledge any contributors who have been removed as authors and ensure you state what contribution any new authors made (if applicable per the journal or book (series) policy). Please ensure these are updated in your manuscript - after approval of the change(s) - as our production department will not transfer the information in this form to your manuscript.

New acknowledgements:

Not applicable

New Disclosures (financial and non-financial interests, funding):

Not applicable

New Author Contributions statement (if applicable per the journal policy):

N.L., A.C., and S.P. designed research; N.L., A.C., I.K., M.K., and S.C. performed research; N.L., A.C., and S.C. analyzed the data; N.L., A.C., and S.P. wrote the paper.

State 'Not applicable' if there are no new authors.

SPRINGER NATURE Springer Nature is one of the world's leading global research, educational and professional publishers, created in May 2015 through the combination of Nature Publishing Group, Palgrave Macmillan, Macmillan Education and Springer Science+Business Media.

Change of authorship request form (pre-acceptance)

	First name	Family name		Signature	Affiliated institute	Date
Co-1st author	Niharika	Loomba	I agree to the proposed new authorship shown in section 4		Vanderbilt Brain Institute, Vanderbilt University, Nashville, TN USA Stephen M. Stahl Centre for Psychiatric Neuroscience, Department of Psychiatry and Behavioral Sciences Northwestern University Feinberg School of Medicine, Chicago IL, USA	4/17/2025
Co-1st author	Anyu	Cao	I agree to the proposed new authorship shown in section 4		Stephen M. Stahl Centre for Psychiatric Neuroscience, Department of Psychiatry and Behavioral Sciences Northwestern University Feinberg School of Medicine, Chicago IL, USA Current affiliation: Neuroscience Graduate Program, University of California, San Francisco, CA, USA	4/17/2025
2nd author	Senna	Charles	I agree to the proposed new authorship shown in section 4		Stephen M. Stahl Centre for Psychiatric Neuroscience, Department of Psychiatry and Behavioral Sciences Northwestern University Feinberg School of Medicine, Chicago IL, USA	4/17/2025
3rd author	Isaac	Kandil	I agree to the proposed new authorship shown in section 4 /**and the addition of my name to the authorship list.**		Stephen M. Stahl Centre for Psychiatric Neuroscience, Department of Psychiatry and Behavioral Sciences Northwestern University Feinberg School of Medicine, Chicago IL, USA	4/17/2025
4th author	Michelle	Kwon	I agree to the proposed new authorship shown in section 4		Stephen M. Stahl Centre for Psychiatric Neuroscience, Department of Psychiatry and Behavioral Sciences Northwestern University Feinberg School of Medicine, Chicago IL, USA	4/17/2025
5th author	Sachin	Patel (CA)	I agree to the proposed new authorship shown in section 4		Stephen M. Stahl Centre for Psychiatric Neuroscience, Department of Psychiatry and Behavioral Sciences Northwestern University Feinberg School of Medicine, Chicago IL, USA	4/17/2025
6th author						

Please use an additional sheet if there are more than 7 authors.

Important information. Please read.

- Please return this form, fully completed, to Springer Nature. We will consider the information you have provided to decide whether to approve the proposed change in authorship. We may choose to contact your institution for more information or undertake a further investigation, if appropriate, before making a final decision.

- By signing this declaration, all authors guarantee that the order of the authors are in accordance with their scientific contribution, if applicable as different conventions apply per

---
